# Genome-Wide Association Study of Wood Anatomical and Morphological Traits in *Populus trichocarpa*

**DOI:** 10.3389/fpls.2020.545748

**Published:** 2020-09-09

**Authors:** Hari B. Chhetri, Anna Furches, David Macaya-Sanz, Alejandro R. Walker, David Kainer, Piet Jones, Anne E. Harman-Ware, Timothy J. Tschaplinski, Daniel Jacobson, Gerald A. Tuskan, Stephen P. DiFazio

**Affiliations:** ^1^Department of Biology, West Virginia University, Morgantown, WV, United States; ^2^Biosciences Division, and The Center for Bioenergy Innovation, Oak Ridge National Laboratory, Oak Ridge, TN, United States; ^3^The Bredesen Center for Interdisciplinary Research and Graduate Education, University of Tennessee, Knoxville, TN, United States; ^4^Department of Oral Biology, College of Dentistry, University of Florida, Gainesville, FL, United States; ^5^Biosciences Center, and National Bioenergy Center, National Renewable Energy Laboratory, Golden, CO, United States

**Keywords:** *Populus*, wood anatomy, leaf morphology, GWAS, networks, lignin

## Abstract

To understand the genetic mechanisms underlying wood anatomical and morphological traits in *Populus trichocarpa*, we used 869 unrelated genotypes from a common garden in Clatskanie, Oregon that were previously collected from across the distribution range in western North America. Using GEMMA mixed model analysis, we tested for the association of 25 phenotypic traits and nine multitrait combinations with 6.741 million SNPs covering the entire genome. Broad-sense trait heritabilities ranged from 0.117 to 0.477. Most traits were significantly correlated with geoclimatic variables suggesting a role of climate and geography in shaping the variation of this species. Fifty-seven SNPs from single trait GWAS and 11 SNPs from multitrait GWAS passed an FDR threshold of 0.05, leading to the identification of eight and seven nearby candidate genes, respectively. The percentage of phenotypic variance explained (PVE) by the significant SNPs for both single and multitrait GWAS ranged from 0.01% to 6.18%. To further evaluate the potential roles of candidate genes, we used a multi-omic network containing five additional data sets, including leaf and wood metabolite GWAS layers and coexpression and comethylation networks. We also performed a functional enrichment analysis on coexpression nearest neighbors for each gene model identified by the wood anatomical and morphological trait GWAS analyses. Genes affecting cell wall composition and transport related genes were enriched in wood anatomy and stomatal density trait networks. Signaling and metabolism related genes were also common in networks for stomatal density. For leaf morphology traits (leaf dry and wet weight) the networks were significantly enriched for GO terms related to photosynthetic processes as well as cellular homeostasis. The identified genes provide further insights into the genetic control of these traits, which are important determinants of the suitability and sustainability of improved genotypes for lignocellulosic biofuel production.

## Introduction

It is of increasing interest to identify the molecular variants underlying adaptive and morphological trait variation in plant populations. Loci highlighted by such analyses have great potential for optimizing the trait of interest through genetic engineering or breeding, thereby producing trees with increased productivity, enhanced abiotic stress tolerance, and/or improved quality of end products. Because of their wide geographic distribution and climatic gradients, large effective population sizes, and high genetic variation, forest trees are excellent model systems for understanding local adaptation and the genetic architecture of complex traits (Neale and Savolainen, 2004; González-Martínez et al., 2006; Neale and Kremer, 2011; Street and Ingvarsson, 2011; Ingvarsson et al., 2016). In this regard, efforts have been made to optimize the ecologically and economically important tree *Populus* for lignocellulosic biofuel production. Vast amounts of genomic and phenotypic resources are available for the genus. Several large-scale genome-wide association studies have identified the underlying genetic architecture related to morphological, physiological, wood property and chemistry, salinity tolerance, and disease resistance traits (Ma et al., 2013; McKown et al., 2014b; Muchero et al., 2015; Zhang et al., 2018; Bdeir et al., 2019; Quan et al., 2019; Jia et al., 2020; Lu et al., 2020). Furthermore, the biology of wood formation, cell wall ultrastructure and composition, and cell wall recalcitrance are fairly well studied (Groover et al., 2010; Wegrzyn et al., 2010; Studer et al., 2011; Du et al., 2013; Porth et al., 2013a; Porth et al., 2013b; Muchero et al., 2015; Porth et al., 2015; Allwright et al., 2016; Du et al., 2016; Escamez et al., 2017; Fahrenkrog et al., 2017; Johnson et al., 2017; Xi et al., 2017; Du et al., 2018; Gandla et al., 2018; Du et al., 2019) and a few studies have also recognized the role of microRNA in controlling tree growth and wood property traits in *Populus* (Quan et al., 2016; Chen B. et al., 2018). However, the genetic architecture underlying wood anatomical traits such as vessel size and density are relatively uncharacterized, despite the importance of these traits for cell wall composition and the overall performance of the tree.

Wood anatomy not only contributes to the structural integrity of the tree, but it is also critical for transport and storage processes (Sperry, 2003; Hietz et al., 2017). Anatomical structures like vessel size and density are related to cell wall structure and composition and wood density. These traits together affect long-distance axial transport of nutrients and hydraulic conductivity. Lignified cell walls and fibers add strength and living parenchyma cells provide radial transport and storage. There are often trade-offs among vessel properties, wood density and hydraulic conductivity (Preston et al., 2006). Wood traits are generally heritable (Carlquist, 2012) and serve as useful traits in phylogenetic analyses (Hietz et al., 2017). Radial variation in wood anatomical properties in the stem affects wood functional traits such as hydraulic conductivity and often scales with tree size and leaf characteristics (Lachenbruch et al., 2011). Genome-wide association studies of wood anatomy traits undoubtedly complement the current understanding of genetic architecture of other structural and functional traits in *Populus* and will serve as an important step for optimizing traits suitable for lignocellulosic biofuels.

Nevertheless, the limitation of power due to sample size in detecting the genetic variants associated with complex traits is a major hurdle in trees and other non-model organisms. The recent technological revolution in genomics has made comprehensive genotyping routine, but challenges remain due to the cost and logistical difficulties inherent in the establishment, management, and intensive phenotyping of large common gardens of trees. Furthermore, SNP loci and candidate genes identified thus far explain only a small proportion of the genetic variation in complex traits in general (Visscher et al., 2017). Genes controlling complex traits do not work in isolation, but instead are interconnected in networks of hundreds to thousands of genes, each of which may contribute incrementally to the variation in complex traits (Boyle et al., 2017). Recently, methods such as multitrait GWAS and meta analyses using summary statistics (e.g., principal components) have become increasingly popular due to their role in enhancing the power of GWAS and the identification of potentially pleiotropic loci (Porter and O’Reilly, 2017). Performing multitrait GWAS directly on raw phenotypic data can enhance power and lead to discovery of novel associations (Chhetri et al., 2019).

Multi-omics network methods can be used to boost the signal of true positives and reduce noise in GWAS results by demonstrating concurrent support for hypotheses from multiple lines of evidence (LOE) (Pendergrass et al., 2013; Verma et al., 2018; Weighill et al., 2019). This can be particularly helpful for studies in which sample size or power are limiting factors (Genovese et al., 2006). Furthermore, LOE methods help identify GWAS hits that are strong gene candidates for experimental validation by examining the gene across multiple contexts in parallel. By considering expression patterns in conjunction with phenotypic associations, genes that have a higher probability of being feasibly validated in lab, greenhouse, and common garden settings can be prioritized.

Here we focus on *Populus trichocarpa*, a targeted species for lignocellulosic biofuel production that has a distribution spanning from northern California to northern British Columbia. Tremendous resources including whole-genome resequencing data, multiple common gardens for association mapping, transcriptome and metabolite data and expression networks are available for this species (Chhetri et al., 2019; Weighill et al., 2019). We present a genome-wide association study of wood anatomical traits for the first time for this species. We also present a GWAS for important morphological traits from the same trees that together with anatomical traits affect overall plant productivity. We complemented the single trait GWAS with multitrait analyses, followed by multi-omic LOE and functional enrichment analyses. Since this study is based on data collected from a common garden in Clatskanie, Oregon this allowed for the direct comparison of GWAS genes identified for some of the same traits from other plantations (Evans et al., 2014; Chhetri et al., 2019).

## Materials and Methods

### Phenotypic Data Collection

**Figure 1:**
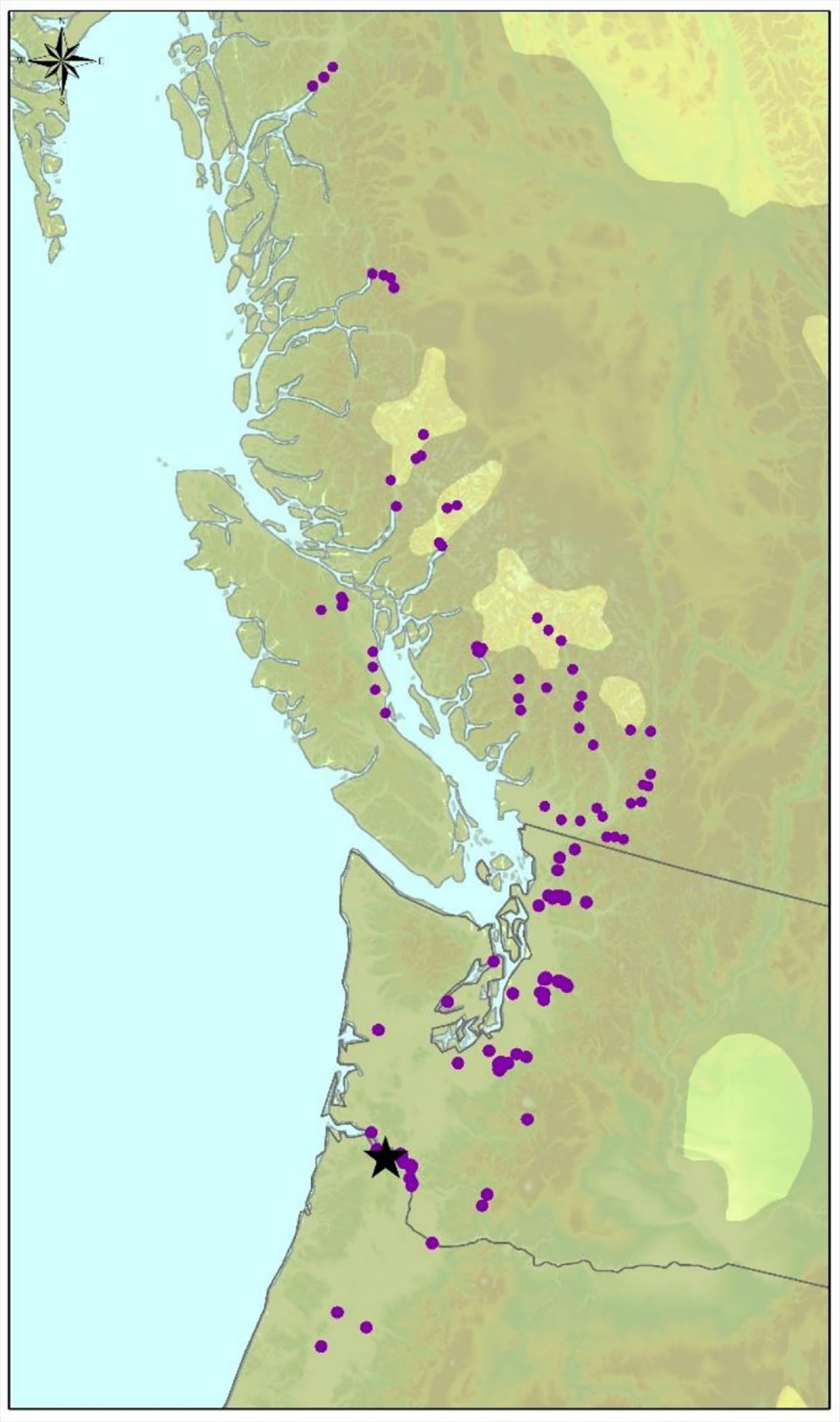
Source locations of 869 P*. trichocarpa* genotypes sampled in this study (purple dots). The trees were grown in a common garden in Clatskanie, Oregon, USA (black star).

Wood anatomical, and morphological trait data were collected from a field trial of 1,100 *P. trichocarpa* genotypes that was established in Clatskanie, Oregon in 2009 (**Figure 1**). These genotypes were previously collected from across the natural range of *P. trichocarpa* from northern California to northern British Columbia and were clonally replicated and planted in a randomized block design with three replicates of each genotype at 2 m x 3 m spacing in the field trial (Slavov et al., 2012; Evans et al., 2014). Phenotyping was performed on clonal replicates for all traits as described in **Table 1**. In some cases replicate observations were recorded for a subset of the genotypes, with the number of replicated genotypes ranging from 59 to 860.

**Table 1:** Broad-sense heritability estimates (*H^2^*) and the number of SNP-trait associations for wood anatomical and morphological traits in *P. trichocarpa*.

Trait	H^2^	Genotypes	Total trees^a^	SNPs with FDR<0.1^b^	Chip_H^2^ (+/- CI)^c^
Wood anatomy					
Early wood vessel area	0.335	489	548 (59)	0	0.542 (+/- 0.545)
Early wood vessel count	0.38	489	548 (59)	0	0.611 (+/- 0.582)
Early wood vessel size	0.392	489	548 (59)	0	0.199 (+/- 0.325)
Intermediate wood vessel area	0.196	570	633 (63)	0	0.475 (+/- 0.300)
Intermediate wood vessel count	0.233	570	633 (63)	0	0.562 (+/- 0.484)
Intermediate wood vessel size	0.218	570	633 (63)	1	0.301 (+/- 0.406)
Late wood vessel area	0.114	557	636 (79)	0	0.373 (+/- 0.359)
Late wood vessel count	0.146	557	636 (79)	0	0.327 (+/- 0.388)
Late wood vessel size	0.22	557	636 (79)	0	0.373 (+/- 0.359)
Morphology					
Diameter (breast height)	0.227	869	2438 (860)	9 (3)	0.929 (+/- 0.278)
Height	0.294	869	2438 (860)	0	0.965 (+/- 0.257)
Leaf area	0.434	676	813 (137)	0	0.87 (+/- 0.349)
Leaf aspect ratio	0.251	676	813 (137)	0	0.439 (+/- 0.320)
Leaf circularity	0.285	676	813 (137)	22 (2)	0.313 (+/- 0.318)
Leaf dry weight	0.452	685	836 (151)	27 (21)	0.99 (+/- 0.325)
Leaf length	0.477	676	813 (137)	0	0.679 (+/- 0.320)
Leaf perimeter	0.459	676	813 (137)	0	0.786 (+/- 0.357)
Leaf wet weight	0.393	687	842 (155)	33 (27)	0.985 (+/- 0.351)
Leaf width	0.386	676	813 (137)	0	0.905 (+/- 0.355)
Maximum petiole diameter	0.426	687	840 (153)	0	0.928 (+/- 0.323)
Minimum petiole diameter	0.249	683	834 (151)	0	0.843 (+/- 0.345)
Petiole length	0.397	685	839 (154)	0	0.987 (+/- 0.335)
SPAD	0.214	687	843 (156)	0	0.232 (+/- 0.282)
Specific leaf area	0.122	667	797 (130)	0	0.038 (+/- 0.318)
Stomatal density	0.477	721	884 (163)	20 (4)	0.597 (+/- 0.294)

a Number of genotypes, with clonal replicates in parentheses.

b Number of SNPs at 10% FDR; Number in parentheses represents SNPs with FDR<0.05.

c Mean chip heritability values for phenotypes, with confidence interval (CI).

### Wood Anatomy

**Figure 2:**
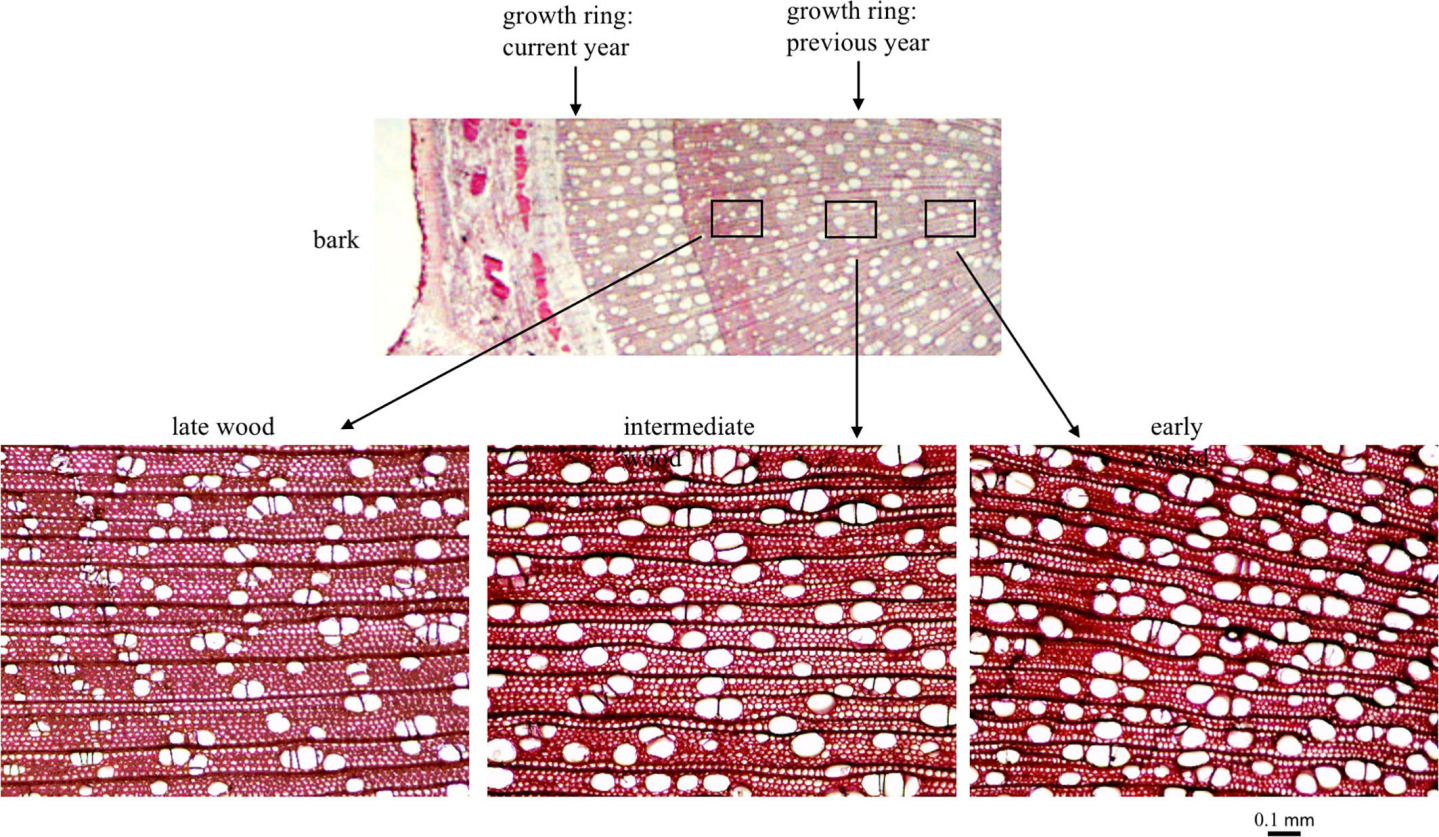
*Populus trichocarpa* wood cross section from the previous year’s growth ring showing variation in early, intermediate and late wood types.

In June 2012, 557 trees were sampled for wood anatomical traits. Wood cores of 5.5 mm diameter were extracted from bark to pith from the southern face of the main trunk of the tree using an increment borer. Free hand cross-sections were made from the previous year’s growth ring and fixed in 70% alcohol. The tissue sections were stained in 1% Safranin O solution for 30 s before preparing the slides for imaging. Images at 100x magnification were taken to sample the early, intermediate and late wood from the growth ring. These images were used for measuring vessel density, size and number (**Figure 2**). All images were processed using the software *imageJ* to extract quantitative measurements—vessel count, density and size (Schindelin et al., 2015).

### Leaf Anatomy

Leaf characteristics were measured for 676–687 trees (**Table 1**). The first and second fully expanded leaf (counting from the apex) were collected from a branch receiving full sunlight. One of the leaves was used for measuring petiole length and minimum and maximum petiole diameter with a caliper and then scanned using a hand-held scanner. The software *imageJ* (Schindelin et al., 2015) was used to estimate leaf area, leaf circularity, leaf length, leaf width, and leaf perimeter. Dry and wet weights were measured for the same leaf and leaf area and leaf dry weight were used to estimate the specific leaf area (SLA). Leaf chlorophyll content (SPAD) was assessed using a SPAD 502 Plus meter (Spectrum Technologies) with an average of three replicate measures on a leaf section. The second leaf was used for measuring the abaxial stomatal density. Clear nail polish was applied to the broadest part of the leaf close to the midrib. A clear piece of tape was then used to capture an imprint of the epidermal leaf surface and stored on microscope slides. The number of stomata in 1 mm^2^ area in four random microscopic fields at 400x magnification were counted.

### Leaf Metabolites

Leaf material was collected from 851 P*. trichocarpa* genotypes. Metabolites were extracted from tissues, measured by GCMS, and peaks were characterized (Weighill et al., 2018). For each sample, a Median Absolute Deviation (MAD) threshold of six was used to remove phenotypic values that were more than six MADs from the population median for that phenotype. In addition, phenotypes with greater than 20% missing data were removed prior to analysis, resulting in a total of 816 metabolite phenotypes. A Benjamini-Hochberg false-discovery rate cutoff of 0.1 was applied to the adjusted *p*-values.

### Wood Composition

The wood cores were ground, processed and analyzed by pyrolysis molecular beam mass spectrometry (py-MBMS) as previously described (Muchero et al., 2015; Weighill et al., 2018). py-MBMS data were TIC normalized to account for variation in sample mass. Phenotypes with > 20% missing data or zeroes were removed prior to analysis (one phenotype, “m/z 76”). For each sample, a Median Absolute Deviation (MAD) threshold of five was used to remove phenotype values that were more than five MADs from the population median for that phenotype, resulting in the removal of an average of 1.49 py-MBMS phenotype values per sample (in total, 625 phenotype values).

### Statistical Analyses

To estimate the genetic control of quantitative traits, broad-sense heritability (*H*^2^) was estimated for all traits using the genotypes with replicate clonal measurements using the following formula:

$$H^{2}=\frac{\sigma_{G}^{2}}{\sigma_{G}^{2}+\sigma_{E}^{2}},$$

where, $\sigma_{G}^{2}$ is genotypic variance due to clonal differences and $\sigma_{E}^{2}$ is environmental variance.

Outliers were removed, and the data were evaluated for normality. Variance components were estimated employing the linear regression model with the *lmer* and *ranef* functions of the *lme4* package implemented in R. Genotype and the position of the tree (i.e. row and column) in the garden was used as a random effect in the model. Error was estimated from the residuals of the model. Genetic correlation between the traits was estimated using the Best Linear Unbiased Predictors (BLUPs) from the same model (**Supplementary Figure S1**). Phenotypic BLUPs were also used for estimating the correlation between the phenotypic traits and 26 geoclimate variables and their first four principal components of the source location of the trees. Genetic correlations were also calculated between phenotypic traits collected at this site to those collected in a clonally replicated trial with the same genotypes in Corvallis, Oregon (Chhetri et al., 2019).

### Genotypic Data

Methods for obtaining genotypic data were as described previously (Weighill et al., 2018; Chhetri et al., 2019). Briefly, whole genome re-sequencing data was obtained from 1,053 trees using Illumina genetic analyzers at the DOE Joint Genome Institute. After removing trees related more closely than first cousins and highly differentiated California trees, 869 trees were left, which were used for all analyses. A genetic relationship matrix was estimated using GEMMA and used as a covariate in the GWAS analyses. Furthermore, SNPs with minor allele frequency ≤0.05 were removed.

### Association Analysis

The tests for statistical association for all phenotypic traits were conducted using the Genome-wide Efficient Mixed Model Association package (GEMMA, Zhou & Stephens, 2012; Zhou & Stephens, 2014), except for the py-MBMS data, which were analyzed using the efficient mixed-model association eXpedited (EMMAX) package (Kang et al., 2010). Phenotypic BLUPs, a genetic relationship matrix and 6,741,160 SNPs were used for the association test. Single trait GWAS was run for 25 phenotypes (**Table 1**). The tested model was:

y=xβ+u+є,

where *y* is an *n*-vector of phenotypic BLUP values, where *n* is the number of individuals tested; *x* is an *n*-vector of marker genotypes, *β* is the effect size of the marker, *u* is an n-vector of random effects that includes a relatedness matrix and *є* is an *n*-vector of errors. We also tested Principal Components as covariates in the model, as described previously (Evans et al., 2014), but we determined that inclusion of PCs resulted in overcorrection, as determined by a preponderance of points below the 1:1 line in Q-Q plots (not shown).

Multitrait GWAS was run for 9 sets of phenotype combinations (**Table 2**). The same procedure was used for selecting trait sets as described in Chhetri et al. (2019). Briefly, traits were combined if there was reason to believe that they could be functionally related. For example, leaf traits were combined with wood anatomy traits due to the expected interactive roles of both sets of traits in water stress tolerance (Sperry, 2000). Multitrait association was conducted with GEMMA using the same model as for single trait associations, except *y* is an *n*×*d* matrix of *d* phenotypes for *n* individuals.

**Table 2:** List of traits used for multitrait associations and significant SNPs identified in *P. trichocarpa*.

Trait combination	Abbreviation	Trait name	PopN^a^	SNPs with FDR<0.1^b,c^
Early wood vessel area, early wood vessel count	EWva_EWvc	wood anatomy multitrait 1	489	0
Early wood vessel area, early wood vessel count, intermediate wood vessel area, intermediate wood vessel count, late wood vessel area, late wood vessel count	EWva_EWvc_IWva_IWvc_LWva_LWvc	wood anatomy multitrait 7	411	0
Early wood vessel area, intermediate wood vessel area, late wood vessel area	EWva_IWva_LWva	wood anatomy multitrait 4	411	0
Early wood vessel area, leaf area, stomatal density	EWva_LA_SD	morphology and wood anatomy multitrait	425	4
Early wood vessel count, intermediate wood vessel count, late wood vessel count	EWvc_IWvc_LWvc	wood anatomy multitrait 5	411	0
Early wood vessel size, intermediate wood vessel size, late wood vessel size	EWvs_IWvs_LWvs	wood anatomy multitrait 6	411	0
Intermediate wood vessel area, intermediate wood vessel count	IWva_IWvc	wood anatomy multitrait 2	570	3
Leaf area, leaf dry weight, leaf length, lead width	LA_LD_LL_LW	leaf morphology multitrait	674	9 (8)
Late wood vessel area, late wood vessel count	LWva_LWvc	wood anatomy multitrait 3	557	3 (3)

a Number of unique genotypes.

b Number of significant SNPs in multi-trait GWAS.

c Numbers in parentheses represent SNPs with FDR <0.05.

We used an FDR cutoff of 0.05 and a more liberal FDR cutoff of 0.1 to identify suggestive associations (Storey and Tibshirani, 2003). For the purpose of summarizing the results, significant SNPs within 10 kb of one another were merged into peaks. Gene models that were closest to significant SNPs were identified based on v3 of the *P. trichocarpa* genome. Annotation information including gene expression level in different plant tissues and annotation of putative gene function was obtained from Phytozome (Goodstein et al., 2012). Percentage of variance explained (PVE) by SNPs was estimated using the formula in Shim et al. (2015).

### Multiple Lines of Evidence (LOE) Analysis

A network was created using five data layers generated from *P. trichocarpa* genetic and phenotypic analyses: pairwise gene coexpression, pairwise gene comethylation, metabolite GWAS, rare variant regional metabolite GWAS, and py-MBMS GWAS. The first four of these layers were previously described (Furches et al., 2019). The py-MBMS GWAS analysis was performed for this study. For each layer, a network was constructed that consisted of gene pairs (nodes) linked by a correlation value or association p-value (edge).

### Coexpression Network

*P. trichocarpa* (Nisqually-1 reference) RNA-seq data was obtained from the DOE Joint Genome Institute Plant Gene Atlas (available from Phytozome at: https://phytozome.jgi.doe.gov/pz/portal.html). For each sample, expression was measured in leaf, stem, root and bud tissues at multiple developmental stages and four different conditions (three nitrogen treatments and an untreated control). Plant cultivation, tissue collection, and RNA extraction and sequencing were previously described in Weighill et al. (2018), as were RNA-seq read trimming, read alignment, TPM calculations, Spearman correlation analysis, and network construction. A correlation value threshold of 0.85 was set as criteria for retention of gene pairs in the coexpression network. As previously demonstrated, this threshold minimized false positives, and the resulting network was significantly different from random (Furches et al., 2019).

### Comethylation Network

*P. trichocarpa* MeDIP-seq data (Vining et al., 2012) was obtained from Phytozome. This data set contained MeDIP-seq reads from ten tissues: bud, callus, male and female catkins, internode, regenerated internode, leaf, phloem, xylem, and root. Read mapping, calculation of transcripts per million reads, Spearman correlation analysis, and network construction were conducted as previously described (Weighill et al., 2018). Gene pairs with correlation values greater than or equal to 0.95 were retained in the comethylation network (Furches et al., 2019).

### Rare Variant Regional Metabolite GWAS Networks

Leaf metabolite data were analyzed using a rare variant regional GWAS analysis as described elsewhere (Weighill et al., 2018). This analysis jointly tested rare SNPs (MAF ≤ 0.01) within each gene’s boundary and the 2-kb up- and downstream flanking regions of that gene using a linear mixed model as implemented by SKAT in RVtest (Zhan et al., 2016). An FDR cutoff of 0.1 was applied to the resulting p-values.

### LOE Scoring of Candidate Genes

The purpose of this LOE method was to explore the networks of genes underlying the traits analyzed in the primary leaf and wood anatomy GWAS analyses to assess support for the functional hypotheses suggested by the gene annotations. For each of the significant (FDR<0.1) gene models identified by the single trait and multitrait GWAS analyses of wood and leaf anatomy traits (hereafter referred to as “anchor genes”), a merged network of anchor genes and their nearest one-hop neighbors in six data layers (including the gene-trait associations detected in this study) was created. Network statistics were calculated, including breadth (the number of layers to which a gene is connected), depth per layer (the number of nodes to which the gene is connected), and total depth. Anchor genes were directly scored based on the number of shared edges in each layer rather than scoring the nearest neighbors of anchor genes. These scores were then used to prioritize genes for further analysis.

### Functional Enrichment of Coexpression Nearest Neighbors

The purpose of this analysis is to test whether functional categories represented in the one-hop networks occur more frequently than expected from a random draw of the same number of genes from the genome. For each anchor gene, two separate Gene Ontology (GO) functional enrichment analyses were performed on positively and negatively coexpressed one-hop neighbors, respectively using the software package Fuento (Weichselbaum et al., 2017). Fisher’s Exact tests were performed for all available namespaces (molecular function, cellular compartment, biological process, aberrant functions) using 100 tries and an FDR adjusted p-value cutoff of p ≤ 0.1.

## Results

### Heritabilities of Wood Anatomy and Morphology Traits

Overall the broad-sense heritabilities estimated for wood anatomy and morphology related traits in this study were low to moderate (**Table 1**). Broad-sense heritabilities for wood anatomy traits ranged from 0.114 for late wood vessel area to 0.392 for early wood vessel size. Late wood anatomy (late wood vessel count, vessel area and vessel size) had low heritabilities compared to other wood traits. For morphology related traits, broad-sense heritabilities ranged from 0.122 for specific leaf area to 0.477 for stomatal density.

Heritability estimates derived from the SNP data (“chip heritabilities”) for wood anatomy traits ranged from 0.199 for early wood vessel size to 0.611 for early wood vessel count whereas for morphology related traits it ranged from 0.038 for specific leaf area to 0.965 for tree height (**Table 1**). Although there was no correlation between broad-sense and chip heritabilities, low chip heritability was generally reflective of low broad-sense heritability and there was a significant correlation between the sample size (number of genotypes used for GWAS) and chip heritability values (r=0.441, *P*=0.021) for the traits measured in this study (**Table 1**). GWAS results for traits with low chip heritabilities should be treated with caution.

### Genetic Correlation of Phenotypic Traits Within and Between the Common Gardens

As expected, most morphological traits measured in the Clatskanie common garden were correlated to each other—leaf traits such as leaf area, leaf length, leaf dry weight, leaf wet weight, petiole diameter and length, leaf perimeter, and leaf aspect ratio were highly significantly correlated to each other and SPAD and stomatal density had low, but significant correlations with most leaf traits (**Supplementary Figure S2**). Similarly, most wood traits were correlated to each other. Wood anatomy traits such as vessel area and size and vessel count within each of the growth ring regions (early, intermediate or late wood indicating a different maturation stages) were correlated to each other. Wood anatomy traits were also significantly correlated to each other across growth ring areas (**Supplementary Figure S2**). Furthermore, some morphological traits such as tree height and diameter were significantly correlated with wood traits such as, early wood vessel size and area, intermediate wood vessel size and count, and late wood vessel size and count (**Supplementary Figure S2**).

Genetic correlation of the same phenotypic traits measured between the Clatskanie and Corvallis common gardens showed that most traits were weak to moderately, but significantly correlated to each other (**Supplementary Figure S3**). Pairwise genetic correlations of tree height (r=0.436, *p*<0.001) and stomatal density (0.345, *p*<0.001) between the sites showed the strongest correlations among all traits compared (**Supplementary Figure S3**).

### Phenotypic Trait Correlations With Climate Variables

As seen for the phenotypic traits in the Corvallis common garden (Chhetri et al., 2019), most phenotypic traits had significant correlations with latitude, and therefore the correlations between the phenotypic traits and other geoclimate variables cannot easily be disentangled. Nevertheless, most morphological traits had significant (although weak) correlations with most geoclimatic variables (**Supplementary Figure S4**). Similarly, wood traits such as late wood vessel area had significant (although weak) correlations with most geoclimatic variables (**Supplementary Figure S4**).

### Genes Identified From Single Trait and Multitrait GWAS

**Figure 3:**
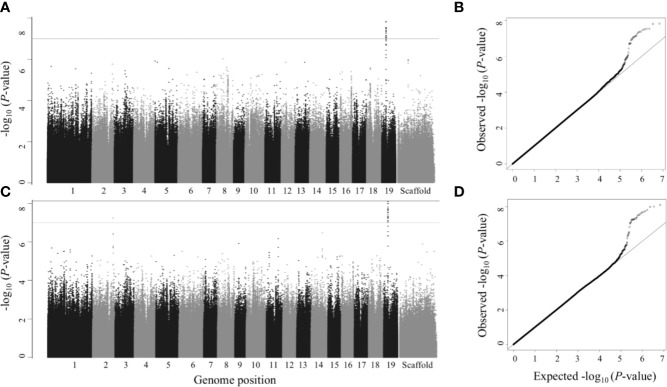
Single trait GWAS – Manhattan (left) and QQ plots (right). Numbers 1 to 19 represent chromosomes; scaffolds are the reads that did not align to any of the 19 chromosomes. **(A**, **B)** Leaf dry weight; **(C**, **D)** Leaf wet weight. SNPs above gray line have *p*<1×10^-7^, which is roughly equivalent to FDR ≤ 0.1 and SNPs above black line (the top line) have *p*<7.417×10^-9^ (the Bonferroni correction threshold), which is roughly equivalent to FDR ≤ 0.05 in this study.

**Figure 4:**
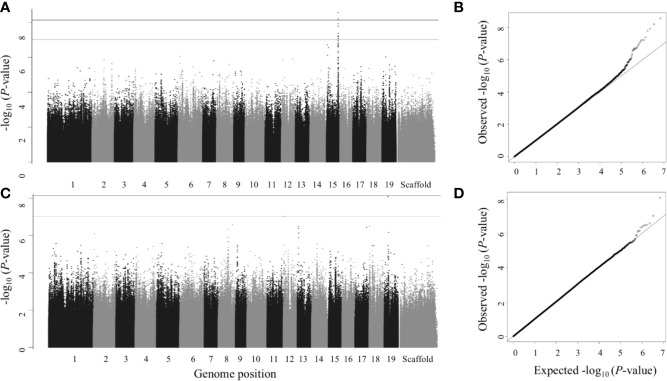
Single trait GWAS – Manhattan (left) and QQ plots (right). Numbers 1 to 19 represent chromosomes; scaffolds are the reads that did not align to any of the 19 chromosomes. **(A**, **B)** Stomatal density; **(C**, **D)** Vessel size (intermediate wood). SNPs above gray line have *p*<1×10^-7^, which is roughly equivalent to FDR ≤ 0.1 and SNPs above black line (the top line) have *p*<7.417×10^-9^ (the Bonferroni correction threshold), which is roughly equivalent to FDR ≤ 0.05 in this study.

We performed single trait GWAS with 6.741 million SNPs for 25 morphological and wood anatomical traits. Fifty-seven SNPs passed the FDR threshold of *p*<0.05 (**Table 1**). However, we identified a total of 112 SNPs that passed suggestive association FDR *P*-value cutoff of 0.1 (**Table 1**, **Figures 3** and **4**, **Supplementary Figures S5** and **S6**, **Supplementary Table S1**). These associated SNPs belonged to 12 separate SNP peaks, and 21 P*. trichocarpa* gene models were identified as nearest neighbors to the significant SNPs (**Table 3**, **Supplementary Table S2**). Percentage of variance explained (PVE) for significant SNPs ranged from 3.30% to 5.65% (**Supplementary Table S1**).

**Table 3:** Genes identified from *P. trichocarpa* single trait GWAS.

Gene model^a^	Trait	P-value^b^	FDR^c^	Functional annotation
Potri.014G117300	Diameter (breast height)	1.95E-08	0.046	Gibberellin 2-beta-dioxygenase 2
Potri.014G117400	Diameter (breast height)	1.88E-08	0.046	similar to MYB family transcription factor
Potri.018G141400	Diameter (breast height)	5.67E-08	0.051	ABC transporter
Potri.019G037900	Intermediate wood vessel size	8.44E-09	0.057	Staufen and related double-stranded-RNA-binding proteins
Potri.005G231500	Leaf circularity	2.46E-07	0.094	Similar to expressed protein in Arabidopsis thaliana; co-ortholog of At1g75400, At1g19680
Potri.005G247200	Leaf circularity	3.27E-07	0.100	PF01535/PF13041 - PPR repeat (PPR)
Potri.005G247400	Leaf circularity	9.20E-08	0.094	similar to prolyl oligopeptidase
Potri.005G247600	Leaf circularity	4.63E-08	0.094	similar to maoC-like dehydratase domain-containing protein
Potri.005G247700	Leaf circularity	1.37E-08	0.047	multi-copper oxidase type 1 family protein
Potri.005G247900	Leaf circularity	9.01E-08	0.094	ubiquitin family protein
Potri.019G042700	Leaf dry weight, leaf wet weight	9.73E-09	0.018	similar to expressed protein in *Arabidopsis* thaliana; co-orthologof At4g27620, At4g27610
Potri.019G042600	Leaf dry weight, leaf wet weight^d^	1.49E-08	0.018	MITOCHONDRIAL OUTER MEMBRANE PROTEIN 25
Potri.002G238700	Leaf wet weight	5.74E-08	0.018	similar to hypothetical protein; co-ortholog of At5g48890
Potri.014G159800	Leaf wet weight	3.32E-07	0.077	Lysosomal acid lipase/cholesteryl ester hydrolase (LIPA)
Potri.015G015200	Stomatal density	1.95E-07	0.084	PF02362 - B3 DNA binding domain
Potri.015G028600	Stomatal density	2.81E-07	0.095	KOG0472 - Leucine-rich repeat protein
Potri.015G113800^e^	Stomatal density	1.62E-07	0.084	PF04578/PF13968 – Protein of unknown function; DUF594, DUF4220
Potri.015G117300	Stomatal density	6.49E-09	0.022	NA
Potri.015G117400	Stomatal density	6.11E-08	0.056	PROTEIN ARGONAUTE 2-RELATED
Potri.015G117500	Stomatal density	2.77E-09	0.019	Peptide-O-fucosyltransferase/GDP-L-fucose:polypeptide fucosyltransferase
Potri.015G117600	Stomatal density	8.31E-08	0.062	zinc finger (C3HC4-type RING finger) family protein;

a Gene models are annotated using v3 of the P. trichocarpa genome.

b SNP p values <1 × 10^-7^.

c FDR at 10% level of significance.

d smallest p-value reported (leaf wet weight).

**Figure 5:**
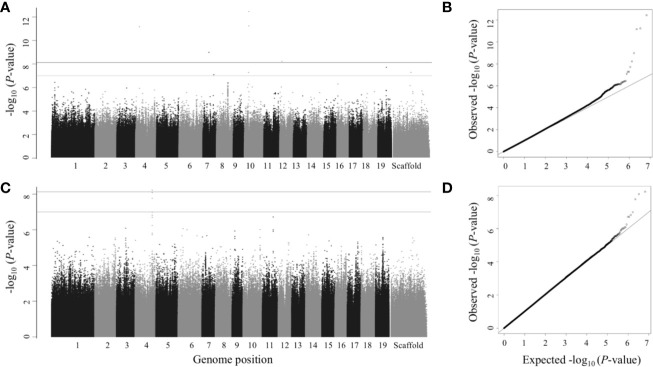
Multitrait GWAS—Manhattan (left) and QQ plots (right). Numbers 1 to 19 represent chromosomes; scaffolds are the reads that did not align to any of the 19 chromosomes. **(A**, **B)** Leaf area, leaf dry weight, leaf length and leaf width; **(C**, **D)** Vessel area and vessel count (late wood). SNPs above gray line have *P*<1×10^-7^, which is roughly equivalent to FDR ≤ 0.1 and SNPs above black line (the top line) have *P*<7.417×10^-9^ (the Bonferroni correction threshold), which is roughly equivalent to FDR ≤ 0.05 in this study.

Multitrait GWAS performed on 9 sets of traits identified 11 SNPs that passed the FDR *p*-value cutoff of 0.05 and 19 SNPs that passed the suggestive association FDR *p*-value cutoff of 0.1. (**Table 2**, **Figure 5**, **Supplementary Figure S7**, **Supplementary Table S3**). These SNPs belonged to 13 separate SNP peaks and were within or close to 13 P*. trichocarpa* gene models (**Table 4**, **Supplementary Table S4**). PVE of these SNPs ranged from 0.01% to 6.18% for the individual traits comprising the multitrait set (**Supplementary Table S3**).

**Table 4:** Genes identified from *P. trichocarpa* multitrait GWAS.

Gene model^a^	Trait	P-value^b^	FDR^c^	Functional annotation
Potri.016G013400	Early wood vessel area, leaf area, stomatal density	3.20E-08	0.054	K15223— upstream activation factor subunit UAF30, SPP27
Potri.004G002600	Early wood vessel area, leaf area, stomatal density	9.57E-09	0.054	UBIQUINOL OXIDASE 4, CHLOROPLASTIC/CHROMOPLASTIC
Potri.015G113800^d^	Early wood vessel area, leaf area, stomatal density	2.22E-08	0.054	Protein of unknown function: DUF594, DUF4220
Potri.006G275800	Intermediate wood vessel area, intermediate wood vessel count	2.37E-10	0.093	LEUCINE-RICH REPEAT-CONTAINING PROTEIN
Potri.001G058100	Intermediate wood vessel area, intermediate wood vessel count	4.15E-08	0.093	similar to expressed protein in Arabidopsis thaliana (co-ortholog of At1g27290)
Potri.004G183900	Late wood vessel area, late wood vessel count	5.91E-09	0.027	SERINE/THREONINE-PROTEIN KINASE
Potri.004G056300	Leaf area, leaf dry weight, leaf length, leaf width	6.88E-12	0.000	Fruit bromelain
Potri.007G061600	Leaf area, leaf dry weight, leaf length, leaf width	1.03E-09	0.002	vacuolar protein sorting-associated protein 35 (VPS35)
Potri.007G099700	Leaf area, leaf dry weight, leaf length, leaf width	8.07E-08	0.059	NB-ARC domain (NB-ARC)/TIR domain (TIR_2)/Leucine rich repeat (LRR_8)
Potri.010G031900	Leaf area, leaf dry weight, leaf length, leaf width	5.23E-08	0.043	similar to RUB-activating enzyme (Ubiquitin activating enzyme E1 like protein)
Potri.010G032600	Leaf area, leaf dry weight, leaf length, leaf width	3.62E-13	0.000	small subunit ribosomal protein S11 (RP-S11, MRPS11, rpsK)
Potri.012G028700	Leaf area, leaf dry weight, leaf length, leaf width	5.90E-09	0.008	Ras suppressor protein (contains leucine-rich repeats)
Potri.019G067300	Leaf area, leaf dry weight, leaf length, leaf width	1.90E-08	0.021	BETA-1,3-GALACTOSYLTRANSFERASE 8-RELATED

a Gene models are annotated using v3 of the P. trichocarpa genome.

b SNP p values <1 × 10^-7^.

c FDR at 10% level of significance.

d Potri.015G113800 is shared between single and multitrait GWAS.

Out of the single trait GWAS for nine wood anatomical traits, we identified only one gene model belonging to intermediate wood vessel size (**Figures 4C, D****)**. A total of three gene models for seven wood anatomy multitrait sets (**Figures 5C, D****)** and three gene models for one morphology and wood anatomy multitrait set (**Figures S6A, B****)** were identified.

### LOE Scores and Rankings

**Figure 6:**
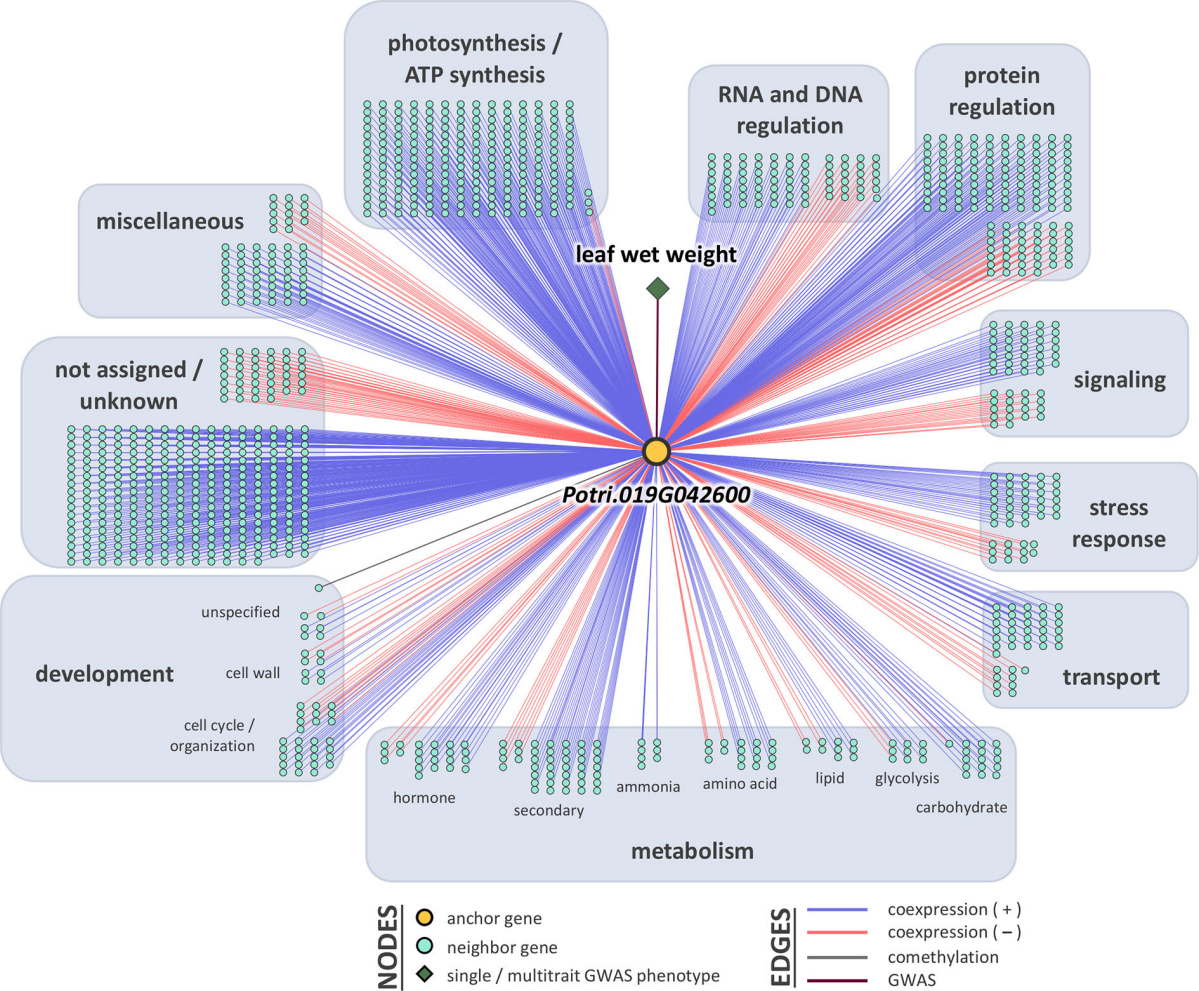
One-hop multi-omic network for the gene Potri.019G042600, which was associated with leaf wet weight.

**Figure 7:**
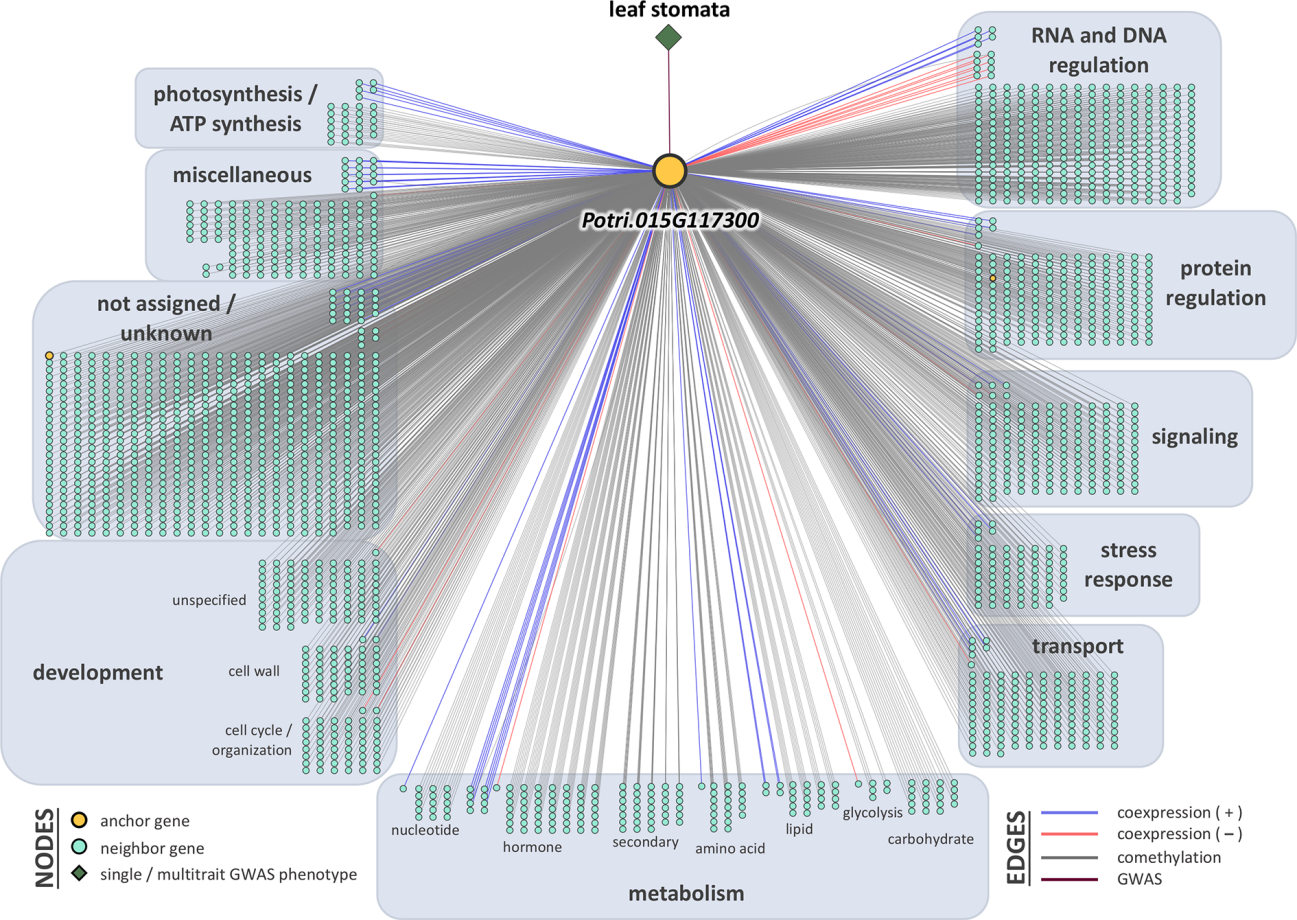
One-hop multi-omic network for the gene Potri.015G117300, which was associated with leaf abaxial stomatal density.

**Figure 8:**
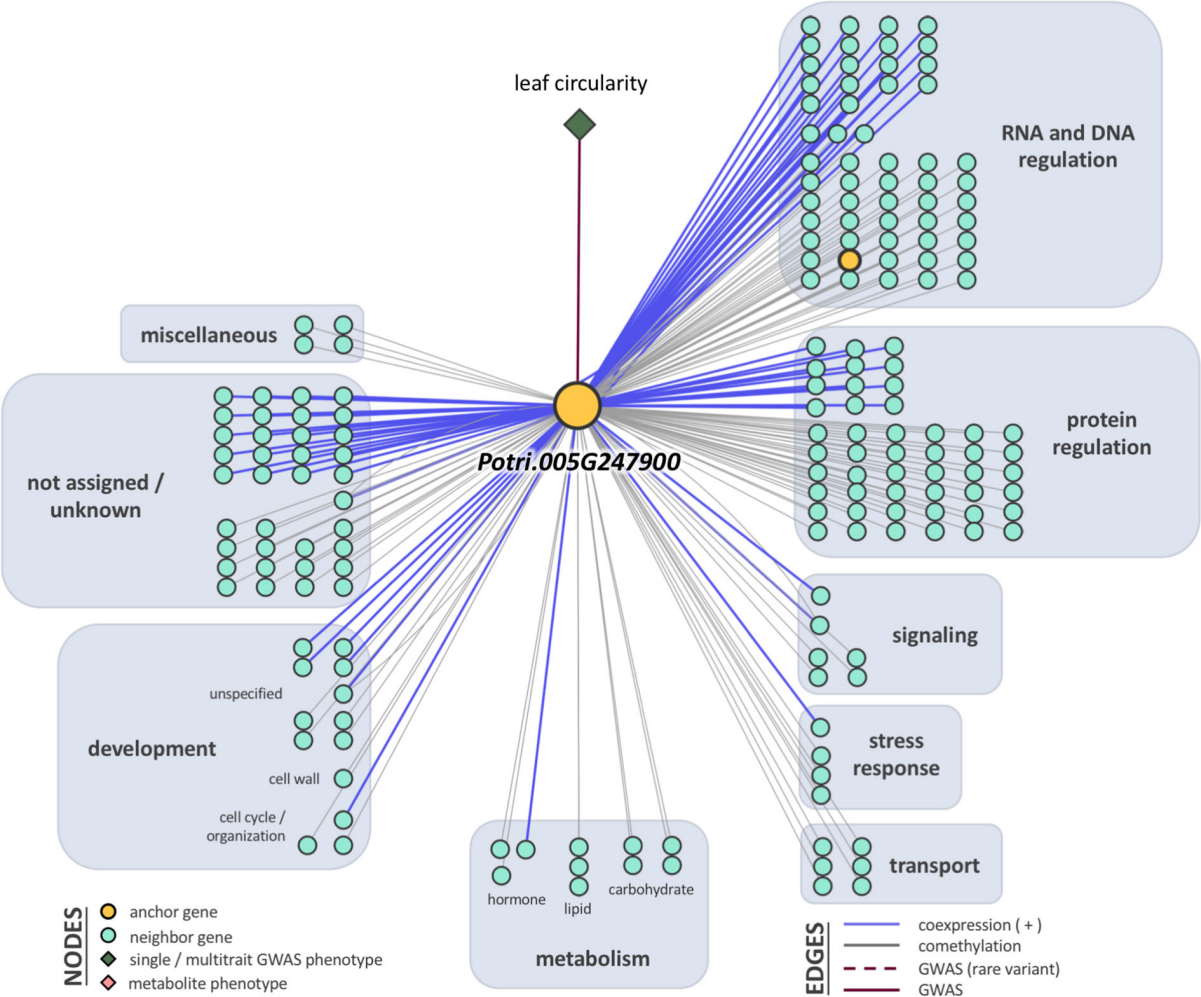
One-hop multi-omic network for the gene Potri.019G067300, which was associated with the multitrait phenotype leaf area, leaf dry weight, leaf length, and leaf width (LA_LD_LL_LW).

Breadth scores ranged from one to four (**Supplementary Table S5**, **Supplementary Figure S8**). Two anchor genes had breadth scores of four: Potri.019G067300 and Potri.010G031900. Both of these genes were associated with the multitrait GWAS for leaf morphology (LA_LD_LL_LW). They have connections to coexpressed and comethylated genes, metabolite, and py-MBMS phenotypes (**Supplementary Tables S6** and **S7**). Two anchor genes had breadth scores of three, both of which were identified through single trait GWAS analysis: Potri.019G037900 (wood anatomy), and Potri.014G159800 (morphology). Total depth scores ranged from one to 2,014. The ranges of layer specific depth scores were: zero to 1,115 for coexpression, zero to 1,914 for comethylation, zero to one for metabolite GWAS, zero to one for RV metabolite GWAS, and zero to one for py-MBMS GWAS. A total of 7630 unique coexpressed and comethylated genes, four metabolites and three py-MBMS GWAS were found to be associated with 33 GWAS (defined as anchor) genes for single and multitrait GWAS (**Figures 6**–**8**, **Supplementary Tables S6** and **S7**, **Supplementary Figures S9**–**S13**).

### Functional Enrichment Results

Thirteen anchor genes had significantly enriched functions among positively coexpressed one-hop neighbors. However, after FDR correction, only five anchor genes had enriched neighborhoods (**Supplementary Table S8**). Seven anchor genes had significantly enriched functions among negatively coexpressed neighbors, but only a single enrichment for one anchor gene remained after FDR correction. Overall there was an abundance of signaling, transport, growth and development, RNA regulation, hormone metabolism, cell wall related and stress related genes for the coexpressed and comethylated genes in the network for all anchor genes (**Supplementary Figure S14**).

### Shared LOE for Single Trait and Multi-Trait Associations

Only one gene, Potri.015G113800, was significant for both a multitrait set (early wood vessel area-leaf area-stomatal density) and for one of the traits making up the set (stomatal density). We also searched for overlaps between the one-hop networks for multi-traits and the individual traits, but again this was the only case where there were shared genes in the one-hop networks (**Supplemental Figure S15**).

## Discussion

### Patterns of Genetic Variation

Broad-sense heritabilities for most traits in this study ranged from 0.2 to 0.4, suggesting that the traits were under moderate genetic control. Heritability estimates for morphological traits were comparable to similar studies (Evans et al., 2014; Mckown et al., 2014; Chhetri et al., 2019). However, some wood related traits and SLA had low heritabilities (<0.2), which may be due to relatively small sample size compared to morphological traits. Low heritability may also be due to micro-environmental variation in the common garden, which can have a strong effect on the growth ring patterns and the anatomical traits studied here. Furthermore, some imprecision in wood trait phenotypes may result from the high throughput preparation method that we were forced to adopt due to the large sample numbers. Wood samples were prepared *via* free-hand sectioning, which results in less uniform images than can be obtained with embedded sections prepared with a microtome. In any case, the results for these low heritability traits should be interpreted with caution.

As reported in previous studies geography plays a major role in shaping adaptive trait variation in *P. trichocarpa*. Correlation of these adaptive traits with latitude can obscure the actual between-trait and trait-climate relationships (Chhetri et al., 2019). Most morphological traits such as tree height and leaf characteristics had strong significant correlations with latitude compared to previous studies (Mckown et al., 2014; Chhetri et al., 2019). In contrast to Chhetri et al. (2019), tree height including diameter at breast height had a very low or no correlation with leaf traits as in McKown et al. (2014c). However, tree height and diameter had significant negative correlations with intermediate and late wood vessel counts and significant positive correlations with vessel size of all maturation stages (early, intermediate and late wood). Interestingly, vessel density was positively correlated with latitude and vessel size was negatively correlated with latitude. It is therefore difficult to deconvolute the indirect effects of latitudinal variation from the direct effects of wood anatomy on productivity in this study.

Further corroboration for the effects of environment shaping these traits were evident when comparing data from the two common gardens. Genetic correlation of the directly comparable traits between the Clatskanie and Corvallis common garden showed very low or no correlations except for stomatal density and tree height (**Supplementary Figure S3**). Furthermore, different loci seem to be controlling phenotypic traits in the two gardens, based on a complete lack of overlap in significant loci discovered by GWAS in the Corvallis, Oregon common garden (Chhetri et al., 2019). This might be due to differential effects of local environments on phenotypes in each of the respective common gardens, or may simply indicate that our analysis is underpowered.

As temperature decreases, and moisture and precipitation increase with latitude, relationships of the phenotypic traits with climate variables corroborates the trait relationships explained above. Leaf characteristics including leaf dry weight and petiole length and diameter had significant positive correlation with latitude, mean annual precipitation and relative humidity, but significant negative correlation with mean annual temperature. Similarly, abaxial stomatal density was negatively correlated with precipitation (**Supplementary Figure S4**). Taken together, southern trees had larger but less dense vessels, smaller leaves with higher abaxial stomatal density and low chlorophyll content, and larger tree height and diameter. Late wood vessel area (similar to the relationship of height and diameter with latitude and temperature) decreased with latitude and increases with temperature.

### GWAS Genes

The GWAS analysis for wood anatomical traits we reported here is the first such study in *Populus*. While we found none to only a few significantly associated or suggestive SNPs for most traits we studied here, we think that this is mainly due to lack of power due to sample size although this is one of the most comprehensive GWAS analyses to date for *P. trichocarpa* in terms of the number of SNPs used. We did not find any gene model controlling more than one trait from our single trait GWAS analyses, but we identified 13 gene models with potential pleiotropic effects controlling sets of morphological and wood anatomical traits (**Table 4**).

### Single Trait GWAS Genes

For single trait GWAS, we identified eight genes belonging to four traits based on 57 SNPs that passed the FDR threshold of 0.05 (**Table 3**). The top five genes identified were for two morphological traits, leaf wet weight (three genes) and stomatal density (two genes).

The gene model identified based on the top GWAS hit for both leaf wet weight and leaf dry weight (**Figure 3**) was Potri.019G042600 which is a mitochondrial outer membrane protein based on PANTHER annotation (https://phytozome.jgi.doe.gov/pz/portal.html). The gene is 6.1 kb downstream of the GWAS SNP (FDR = 0.015, *p*-value = 7.18 × 10^-9^). Outer membrane proteins in mitochondria (and chloroplasts) play a role in important cellular functions such as protein import and ion exchange (Lee et al., 2014). The Arabidopsis homolog of this gene, AT5G17170 encodes rubredoxin family protein located in the plant chloroplast and is involved in electron transport and metal ion binding activity in Arabidopsis (https://www.arabidopsis.org/). Expression of rubredoxin family protein in a grass, *Puccinellia tenuiflora* (*PutRUB)* was shown to be induced by abiotic stresses due to NaCl, NaHCO_3_, CuCl_2_, and H_2_O_2_ suggesting a role of *PutRUB* in stress tolerance. Overexpression of *PutRUB* in Arabidopsis plants under stressed conditions showed an increase in seedling weight in overexpressed plants compared to wild type (Li et al., 2016). The GWAS gene has a breadth score of 2 and a total depth score of 1,116 (3^rd^ highest among all single and multitrait anchor genes, **Figure 6**, **Supplementary Table S5**). It is highly expressed in late winter apical bud and fully open bud, stem, young and first fully expanded leaf and stem treated with ammonia, nitrate and urea (**Supplementary Table S7**). The anchor gene is coexpressed with 1,115 other genes (**Figure 6**) of which 915 are positively and 200 are negatively coexpressed. The coexpressed genes have functions related to photosystem I and II in photosynthesis (155 genes), oxidation reduction reaction (26), RNA regulation and binding (62), signaling (53, including light, G-protein, calcium, receptor kinases and sugar and nutrient physiology), abiotic and biotic stresses (25), transport (36), glycolysis (11), and hormone metabolism (25). All of the top 10 coexpressed genes are related to the photosynthesis electron transport chain including Photosystem I and Photosystem II complexes (**Supplementary Table S6**). Similarly, Potri.019G042600 showed significant functional enrichment for GO term Biological Processes (photosynthesis and respiration related) with functional annotations including ATP synthesis, oxidation reduction, glycolysis and photosynthesis. The gene also showed enrichment for GO term Molecular Function that included hydrolase, oxidoreductase, calcium ion binding, antioxidant activity and proton transport (**Supplementary Table S8**). In sum, network and functional enrichment analyses suggest that the function of this GWAS gene is related to photosynthetic and respiration processes and responses to biotic and abiotic stresses. One of the possible mechanisms by which plants respond to abiotic stresses, especially nutrient and water stress, is by changing morphology (e.g, leaf area and weight) and physiological processes (e.g., respiration) (Rymaszewski et al., 2017), thereby raising the expectation for coordinated responses of genes related to photosynthesis, oxidation reduction reactions, glycolysis, signaling, hormone metabolism, and stress responses, along with those controlling leaf morphology.

Potri.015G117500 was the top gene associated with stomatal density (*p*-value = 2.77 × 10^-9^, **Figures 4A, B****)**. The top GWAS SNP is a synonymous substitution harbored within the gene. Potri.015G117500 encodes peptide-O-fucosyltransferase, which belongs to a family of genes involved in post-translational modification (Lira-Navarrete et al., 2011). The gene had a breadth score of 1 and the total depth score of 17 (comethylated with 17 other genes, **Supplementary Table S5**). Notable comethylated genes include a HSP20-like chaperone super family protein (Potri.009G153200), photosystem II oxygen evolving PsbQ complex subunit protein (Potri.011G031300) and farnesylcysteine lyase (Potri.007G101700) that indirectly has a negative regulatory effect in the ABA mediated signaling pathway in Arabidopsis (Huizinga et al., 2010). Another coexpressed gene, Potri.019G006900 encodes kunitz trypsin inhibitor and is involved in apoptosis during plant-pathogen interactions and responding to salt stress and salicylic acid. Kunitz trypsin inhibitors are known to have a function in pest resistance in poplar (Major and Peter Constabel, 2008). Similarly, another comethylated gene, Potri.001G045500, encodes cinnamoyl CoA reductase which is involved in lignin biosynthesis (Lacombe et al., 1997; Kawasaki et al., 2006). Lignin biosynthetic genes are known to affect growth and defense mechanisms in plants (Xie et al., 2018).

In *Arabidopsis* the O-fucosyltransferase SPINDLY (*SPY*, similar to the protein encoded by Potri.015G117500) affects DELLA and associated regulators such as brassinazole-resistant1 (*BZR1*) phytochrome-interacting-factor3 (*PIF3*) and *PIF4* in the brassinosteroid- and light-signaling pathways (Zentella et al., 2017). DELLA proteins are important integrators of multiple signaling pathways in flowering plants. DELLAs are repressors of phytohormone (GA) signaling, master growth repressors that restrict plant growth by affecting genes in cell division, expansion and differentiation (Zentella et al., 2007). In *Arabidopsis* O-fucosyltransferase modifies the DELLA protein RGA (repressor of *ga1-3*). The GA-deficient mutant *ga1-3* is a nongerminating, extreme dwarf that flowers late and produces male-sterile flowers. This protein is also known to be involved in cell-to-cell adhesion (deficiency in pectin biosynthesis pathway leading to loss of cell adhesion) together with *GAUT*8 (QUASIMODO1, see below) in *Arabidopsis* (Verger et al., 2016). McKown et al. (2014a) identified a similar gene, Potri.004G059000, encoding BRASSINOSTEROID-INSENSITIVE 2 for stomatal density based on the GWAS using a 34K SNP array data for 464 P*. trichocarpa* trees. BRASSINOSTEROID-INSENSITIVE 2 potentially affects the brassinosteroid signaling pathway and the regulation of stomatal development (Gudesblat et al., 2012; Khan et al., 2013). Brassinosteroids also play important role in plant growth, development and stress responses (Nolan et al., 2020).

The other top GWAS hit for stomatal density was within a gene model Potri.015G117300, a gene of unknown function (**Figures 4A, B****)**. The anchor gene has a breadth score of 2 and a total depth score of 2014 (highest breadth score among all single and multitrait anchor genes, **Figure 7**, **Supplemental Table S5**). The gene is comethylated and coexpressed with 1914 and 100 other genes, respectively, that encode proteins related to hormone signaling, stress response and transport functions. The anchor gene is coexpressed or comethylated with the genes related to diverse functions including photosynthesis (22), cell wall (44), development (84), glycolysis (6), hormone metabolism (56), UDP glucosyl and glucoronyl transferases (6), redox (12), signaling (153, including receptor kinase, MAP kinase, light, sugar, G-proteins and calcium), stress (64, including abiotic and biotic, PR-proteins, respiratory burst), transport (123). For example, Potri.015G117300 is comethylated with two other genes, Potri.001G139700 and Potri.005G109300, both of which encode for plasma membrane proteins that belong to the subfamily of plant aquaporins (AQPs) that play important roles in plant physiological processes (Chaumont and Tyerman, 2014). Membrane proteins have been shown to have an involvement in regulating plant-water homeostasis and abiotic stress responses (Sade et al., 2009). Similarly, the anchor gene is comethylated with Potri.005G123200, that encodes for Sec14p-like phosphatidylinositol transfer family protein that has important role in lipid signaling pathways in plants (Huang et al., 2016). Another comethylated gene, Potri.005G134900, encodes a LATERAL ORGAN BOUNDARIES (*LOB*) domain protein. Proteins in this family play a role in organ development and stress responses (Grimplet et al., 2017), and also in regulating the expression of *VND7*, a master regulator of tracheary elements in plants (Ohashi-Ito et al., 2018). Overexpression of *VND7* represses *KNOX* gene activity, which in turn is responsible for meristem and organ development including the development of leaf primordia in plants (Hake et al., 2004) The anchor gene is coexpressed with germin-like (GLP) protein 1 (Potri.013G141900). *GLPs* are involved in a wide range of functions including growth and development, and biotic and abiotic stress responses (Dunwell et al., 2008). Another coexpressed gene, Potri.009G140900, encodes Small Auxin-Up RNA (SAUR)-like protein is involved in plant growth and development (cell division in particular) and the expression of the gene is regulated by auxin (Markakis et al., 2013). The coexpressed gene, Potri.010G112800 encodes auxin efflux carrier family protein, PIN-FORMED 3 (*PIN3*) which plays a role in seedling elongation and shade avoidance in Arabidopsis (Keuskamp et al., 2010). Another coexpressed gene, Potri.011G067500, encodes CBL-interacting protein kinase which is involved in drought tolerance mechanisms and the regulation of stomatal aperture (Wang et al., 2016).

Stomatal density in leaves facilitates gas exchange, and variation in the density of stomata can have a significant effect on overall plant fitness as it can change the dynamics of photosynthetic rate, respiration and growth (Franks and Farquhar, 2007; Franks and Beerling, 2009). Our GWAS and network analyses highlight the importance of genes involved in hormonal signaling and stress responses. Other recent studies in *Populus* have suggested a relationship between stomatal patterning and the trade-off between growth and disease susceptibility (and presence of defense compounds) (McKown et al., 2014a; McKown et al., 2019).

### Multitrait GWAS Genes

We identified a total of seven genes based on the 11 SNPs that passed an FDR threshold of 0.05 in the multitrait GWAS, of which 6 genes were detected for the leaf morphology multitrait, (leaf area, leaf dry weight, leaf length and leaf width) and 1 gene was detected for wood anatomy multitrait (late wood vessel area and vessel count) (**Figure 5**)

The first anchor gene for the leaf morphology multitrait set was Potri.019G067300 (breadth score 4, total depth score 395, FDR=0.021, *p*-value=1.9 x 10^-8^; **Figures 5A, B** and **8**, **Supplementary Table S5**). The gene encodes a protein related to galactosyltransferase-8 (*GAUT*8). The gene is comethylated with 392 other genes, positively coexpressed with one other gene, and related to one metabolite and linked to a py-MBMS (wood chemistry) GWAS. The anchor gene is comethylated with genes with various functions including cell wall (11 genes), growth and development related (11), glycolysis and Krebs cycle related (6), photosystem I and II related (4), oxidation reduction related (6), RNA regulation & binding (37), signaling (31, includes receptor kinase, light, photosynthesis, G-proteins and calcium signaling), stress (16, abiotic such as drought/salt and heat, biotic such as PR-proteins, respiratory burst), and transport (15) (**Figure 8**). The coexpressed gene Potri.006G049600 encodes concanavalin A-like lectin protein kinase family protein that is involved in amino acid phosphorylation. Lectin receptor-like kinases (*LecRLK)* are known to regulate abiotic and biotic stresses, and development in plants (Vaid et al., 2013; Yang et al., 2016; Labbe et al., 2019). A comethylated gene with a similar function as the anchor genes is Potri.006G137100, which encodes a pectin lyase-like superfamily protein that functions in pectin methylesterase (*PME*) activity. *PMEs* are involved in regulation of plant growth and development *via* structural modeling of the cell wall and also play an important role in heat stress tolerance (Wu et al., 2018). Another gene with a similar function, Potri.004G111000 (*GAUT*9) was detected for the same multitrait GWAS from the Corvallis common garden (Chhetri et al., 2019). *GAUT*8 and *GAUT*9 belonged to the same clade B1 in a phylogenetic tree based on the *GAUT* protein family of *Arabidopsis thaliana* and *P. trichocarpa* (Biswal et al., 2018). *GAUT*9 affects leaf size in *Populus deltoides* (Chhetri et al., 2019) whereas *GAUT*8 (also known as QUASIMODO1) has high expression in stems in *Arabidopsis* (Caffall et al., 2009) and is thought to be involved in cell wall pectic homogalacturonan (HG) and xylan biosynthesis (Orfila et al., 2005; Verger et al., 2016). Genes encoding glycosyltransferases, a large family of enzymes mainly involved in biosynthesis of polysaccharides and glycoproteins in the plant cell wall (Hansen et al., 2012), were also detected in single trait GWAS for stomatal density in this study (**Table 3**).

Another gene associated with the leaf morphology multi-trait set was Potri.010G031900 (FDR = 0.043, *P*-value = 5.23 x 10^-8^) is coexpressed and comethylated with 12 and 67 other genes, respectively (breadth score 4, total depth score 81, **Figures 5A, B**, **Supplementary Table S5**). The gene encodes E1 C-terminal protein, which is a RUB-activating enzyme that indirectly triggers auxin response by interacting with the cullin *AtCUL1* in Arabidopsis (Del Pozo and Estelle, 1999). The anchor gene is coexpressed or comethylated with genes with diverse functions including signaling (9, including G-proteins, MAP kinase, receptor kinase and calcium), RNA regulation (9), transport (3), stress (5, abiotic- drought/salt and heat, biotic—PR-proteins), hormone metabolism (4, auxin 2, ABA 2), and lipid metabolism (2) (**Supplementary Figure S13**).

A few of the top coexpressed genes in the network are related to the RING/U-box superfamily protein (e.g., Potri.018G064400). U-box (PUB) proteins have diverse functions in plants, including hormonal, defense and abiotic stress responses (Yee and Goring, 2009). RNA binding protein (Potri.019G017900) and mitogen-activated protein kinase kinase 3 (*MKK3*, Potri.001G345500)—*MKK3* belong to a bigger group of mitogen-activated protein kinases (*MAPKs*) that play a role in activating downstream hormonal signaling including auxin, jasmonic acid, salicylic acid, brassinosteroid, ethylene and ABA signaling targets in plants (Jagodzik et al., 2018). The gene is also comethylated with genes with similar roles such as Potri.009G015800 encoding IQ-domain 5 (IQD5) that functions in calmodulin binding. Calmodulin and calmodulin-like proteins are involved in responding to abiotic stresses in plants (Zeng et al., 2015; Badmi et al., 2018). There are other comethylated genes in the network with functions in stress response, and growth and development. Potri.009G001200 encodes RING-H2 group F2A (*RHF2A*) protein. RING-H2 belong to a group of E3 ubiquitin protein complexes and are involved in defense response, regulation of growth and development, and apoptosis (Guzmán, 2012). Potri.008G010800 encodes ABA-responsive element binding protein 3. Potri.002G152100 encodes phospholipase D beta 1 (*PLDBETA1*). PLD functions in regulating plant growth and development, hormonal signaling and stress responses (Li et al., 2007). The anchor gene is also associated with a metabolite, caffeoyl-quercetin glycoside and one py-MBMS GWAS, m/z 414). Quercetin glycosides are known to play a role in osmotic adjustment during drought stress (Tschaplinski et al., 2019). Caffeoyl-quercetin glycoside was found to be associated with Potri.001G411800 (EF-hand Calcium-Binding Domain protein) in a multitrait GWAS for leaf area, stomatal density and carbon isotope composition in a previous study (Chhetri et al., 2019). Ethylene and ABA play important roles in stress responses with prominent effects in plant tissues (Cramer et al., 2011). Reduction in Leaf water potential and thus leaf wet weight is possible due to abiotic (e.g. drought) and biotic stress (e.g. pathogen) (Pandey et al., 2017). Drost et al. (2015) provided evidence for the effect of *PtARF1* on leaf morphology in a hybrid population of *P. deltoides* and *P. trichocarpa* that is mediated by auxin signaling such that the PIN polarization in leaf cells possibly plays a role in differential expression of *PtARF1*, ultimately causing the expansion of leaf in the length direction with the decrease in leaf width in *P. trichocarpa* and vice versa in *P. deltoides* (Kalluri et al., 2007). Various signaling genes including G-protein signaling (Lease et al., 2001), and hormonal signaling genes (McSteen and Zhao, 2008) are known to have an effect on plant growth and development. Calcium signaling regulates the function of stress related genes ultimately affecting the plant phenotype (Tuteja and Mahajan, 2007).

### Multitrait GWAS Genes: Wood Anatomy

The gene model Potri.001G058100 was associated with the wood anatomy multitrait (**Figure 5**). This gene has no known function but is coexpressed and comethylated with 9 and 972 other genes (breadth score 2, total depth score 981, 4^th^ highest depth score among all single and multitrait GWAS genes), respectively (**Supplementary Tables S5** and **S9**). The gene is comethylated with 3 RNA binding genes (Potri.009G152900, Potri.013G094400, Potri.017G063700) that are involved in biogenesis of small RNA in Arabidopsis (Eamens et al., 2009; Eamens et al., 2012). Small RNAs are known to regulate the expression of genes related to growth and development, and wood formation in plants (Tang et al., 2016; Chen C. et al., 2018). The anchor gene is coexpressed or comethylated with diverse genes that have functions in transport (61 genes), biotic and abiotic stress tolerance (35), signaling mechanism (84, hormonal, receptor kinase, light and calcium, sugar and G-protein signaling), cell wall related (20), developmental mechanisms (39), hormone metabolism (25) and RNA regulation (106) (**Figures 4C, D**; **Supplementary Table S9**). Notable top 10 coexpressed genes include Potri.001G043100 that encodes Chaperone DnaJ-domain superfamily protein which is a heat shock protein with a function in stress tolerance, Potri.002G169400 (encodes RCAR1, regulatory components of ABA receptor), Potri.009G033300 (encodes SCARECROW-like 14, *SCL14*) and Potri.017G134800 (encoding zinc finger protein 7, *ZFP7*). *SCL14* is a member of GRAS family of transcription factors that interacts with the TGA2 transcription factors and affects the transcription of stress-responsive genes (Fode et al., 2008). *ZFP7* is a member of *C2H2* zinc finger protein related to *ZFP3*. *ZFP3* and related *ZFP* proteins including *ZFP7* acts as a negative regulator of ABA signaling during seedling development, affect vegetative growth and regulate light signaling in Arabidopsis (Joseph et al., 2014). ABA receptors are involved in transmitting ABA signals that allow plants to respond to drought and other abiotic stresses (Fan et al., 2016; Duarte et al., 2019). The anchor gene is comethylated with Leucine-rich repeat protein kinase family protein (Potri.019G001800) that functions in ATP binding activity and regulating hormonal signaling and stress response in plants (Máthé et al., 2019). Taken together, this suggests a role of this gene in growth and development including wood formation by maintaining cellular homeostasis, signaling and stress response.

### Comparison With Previous Studies

We compared our GWAS results with previous GWAS studies for similar traits in *P. trichocarpa* (Wegrzyn et al., 2010; Porth et al., 2013a; Porth et al., 2013b; Evans et al., 2014; McKown et al., 2014b; Chhetri et al., 2019) and other *Populus* species including *P. deltoides* (Fahrenkrog et al., 2017), *P. euphratica* (Ma et al., 2013; Jia et al., 2020) and *P. tomentosa* (Quan et al., 2016; Chen B. et al., 2018; Quan et al., 2019; Lu et al., 2020). We found very minimal overlap in the genes and/or gene families detected. Ma et al. (2013) identified multiple gene families that control adaptation in saline environments. Similar to their results, heat shock protein families and some ABA signaling proteins were found in the co-expression networks in our study. Jia et al. (2020) identified 82 genes related to seed salinity tolerance in *P. euphratica*, including a zinc finger protein similar to one that we detected for leaf abaxial density that regulates gas exchange in plants. Lu et al. (2020) identified 23 genes involved in three growth and six wood property traits and Quan et al. (2019) identified 203 lignin biosynthetic genes, 81 TF genes, 36 microRNAs and 71 long non-coding RNA genes for 10 growth and wood property traits in the same *P. tomentosa* population. However, these studies have no apparent overlap in gene functions for similar morphology or wood property traits in our study.

Comparing our findings with previous *P. trichocarpa* studies we identified some non-overlapping gene models with similar functions in shared gene families. For example, we identified a gene Potri.019G067300 in a multi-trait GWAS for leaf morphology that encodes the GAUT8 protein. In a previous multi-trait GWAS study of leaf morphology at a different site, we identified GAUT9 (Potri.004G111000), which affects leaf size and is involved in pectin and xylan biosynthesis (Chhetri et al., 2019). Likewise, we identified a gene (Potri.005G247700) encoding “SKU5 similar 5” (sks5) that functions in oxidoreductase activity and copper ion binding in this study. Porth et al. (2013a) also identified a gene with similar function for soluble lignin (Potri.001G000500) encoding “SKU5 similar 12” (sks12) based on 34K SNP genotyping array data. McKown et al. (2019) identified several genes related to plant growth, physiological processes, biotic and abiotic stresses, and defense and immunity for abaxial and adaxial stomata related traits (stomatal density and pore length). We did not find any of the genes they identified in their study, despite analyzing similar traits here. The discrepancies in the gene models identified across the studies may be due to a) differential environmental effects on the traits due to plantation sites, b) traits measured in different seasons of the year and different years were subjected to different environmental conditions, c) variability in the microenvironment within the sites as reflected by the different broad-sense heritability values, d) differences in the developmental stages of the traits across the studies, e) difference in the genotypes and the sequence data types (e.g. SNP array vs whole genome sequence data) and f) the lack of power in this and previous studies. Furthermore, we were not able to replicate any significant SNP hits or the gene models for the same traits from another *P. trichocarpa* common garden in Corvallis, Oregon in our study, although several single traits such as stomatal density, petiole diameter and tree height and the multitrait set containing leaf area, leaf dry weight, leaf length and leaf width were common between the two common gardens, and measurements were performed using the same methods. This might very well reflect the differential influence of the environment on genotypes in the two populations. Nevertheless, we believe that our comprehensive GWAS study with genome-wide sequencing data highlights the underlying genes controlling adaptive traits in *P. trichocarpa* and complements the findings from other similar studies. Moreover, we have reported here potential genes underlying complex wood anatomy traits in *Populus* for the first time.

## Conclusion

We presented here the first comprehensive GWAS for wood anatomical traits in *Populus* that provides insights into the type of genes controlling structural and functional properties important for plant development, function and stress tolerance. We complemented this with GWAS for important morphological traits that have functional relationships with wood anatomical traits. As shown in a previous *P. trichocarpa* GWAS study in another common garden (Chhetri et al., 2019), we identified additional genes with multitrait GWAS in this study as well. Furthermore, the multitrait sets formed based on the genetic correlations and functional relationships of the traits within and among the wood anatomical and morphological traits provided insight into pleiotropic genes controlling these traits. Some of the genes we identified in this study had no known functions. However, the LOE network analyses we performed here provided additional information not only for suggesting the role of the unknown genes, but also added an extra layer of support for the genes with known functions. Genes identified here, especially the genes controlling wood anatomical traits, can be good targets for biotechnological approaches toward optimizing wood traits for biofuel production. However, as is the case with most GWAS studies, despite the use of large scale whole genome resequencing data and much broader sampling compared to most previous studies in trees, a very small percent of the variation in the traits were explained by the significant SNPs. It is possible that we may simply be underpowered to detect SNPs with small effect sizes, given that we only detected a handful of genes or none for most of the complex traits that might potentially be controlled by hundreds or thousands of genes (Boyle et al., 2017). A more robust approach to GWAS is required, with large and homogeneous sampling from across the range of distribution. Alternatively, more populations with controlled structure can be used to take full advantage of the range of variation present while explicitly controlling for underlying structure, as is the case in nested association mapping (Yu et al., 2008). Moreover, none of the genetic variants detected in the GWAS from another common garden were validated in this study. This may be because of the differential effects of the environment between the common gardens, which is somewhat reflected by very low correlation of the traits between the two common gardens. Furthermore, additional variation due to the difference in the timing (different years) of data collection might have influenced the GWAS study. Nevertheless, by integrating data from multiple sources we were able to apply a LOE approach that provided further corroboration for relatively weak associations, while providing insights into gene function.

## Data Availability Statement

Populus trichocarpa genome sequence, annotation, and Gene Atlas expression and methylation data sets are available on Phytozome (http://phytozome.jgi.doe.gov). Populus trichocarpa variant data (DOI 10.13139/OLCF/1411410) is available from https://doi.ccs.ornl.gov/ui/doi/55.

## Author Contributions

HC, AF, DM-S, AW, DK, PJ, and DJ performed statistical and computational analyses. AH-W and TT performed metabolite analyses and analyzed the data. DJ, GT, and SD oversaw the study. HC, AF, and SD drafted the manuscript. All authors contributed to the article and approved the submitted version.

## Funding

This research was supported by the Center for Bioenergy Innovation (CBI) and the Bioenergy Science Center. CBI is supported by the Office of Biological and Environmental Research in the DOE Office of Science. This manuscript has been coauthored by UT-Battelle, LLC under Contract No. DE-AC05-00OR22725 with the U.S. Department of Energy. The United States Government retains and the publisher, by accepting the article for publication, acknowledges that the United States Government retains a non-exclusive, paid-up, irrevocable, worldwide license to publish or reproduce the published form of this manuscript, or allow others to do so, for United States Government purposes. The Department of Energy will provide public access to these results of federally sponsored research in accordance with the DOE Public Access Plan (http://energy.gov/downloads/doe-public-access-plan). The work conducted by the U.S. Department of Energy Joint Genome Institute is supported by the Office of Science of the U.S. Department of Energy under Contract No. DE-AC02-05CH11231. This research described herein was supported by an award of computer time provided by the INCITE program and used resources of the Oak Ridge Leadership Computing Facility (OLCF) at the Oak Ridge National Laboratory. This work was authored in part by Alliance for Sustainable Energy, LLC, the manager and operator of the National Renewable Energy Laboratory for the U.S. Department of Energy (DOE) under Contract No. DE-AC36-08GO28308. Funding was provided by U.S. Department of Energy Office of Energy Efficiency and Renewable Energy Bioenergy Technologies Office.

## Conflict of Interest

The authors declare that the research was conducted in the absence of any commercial or financial relationships that could be construed as a potential conflict of interest.
