# Supplementary material for: Genome-Wide Association Study of Wood Anatomical and Morphological Traits in Populus trichocarpa
Source: Front Plant Sci. 2020 Sep 9;11:545748. doi: 10.3389/fpls.2020.545748 (PMC7509168; doi:10.3389/fpls.2020.545748)
Supplement: Supplementary file 2 [file Image_1.pdf]

Supplementary Material

Genome-wide association study of wood anatomical and morphological traits in *Populus trichocarpa*

Hari B. Chhetri, Anna Furches, David Macaya-Sanz, Alejandro Riveros Walker, David Kainer, Piet Jones, Anne E. Harman-Ware, Timothy J. Tschaplinski, Daniel Jacobson, Gerald A. Tuskan[2], Stephen P. DiFazio[*]

*** Correspondence:**
Stephen DiFazio
spdifazio@mail.wvu.edu

Suggested Citation:

Chhetri, HB, Furches A, Macaya-Sanz D, Walker AR, Kainer D, Jones P, Harman-Ware AE, Tschaplinski TJ, Jacobson D, Tuskan GA and DiFazio SP (2020). Genome-Wide Association Study of Wood Anatomical and Morphological Traits in *Populus trichocarpa*. Front. Plant Sci. 11:545748. doi: 10.3389/fpls.2020.545748

1 Supplementary Figures

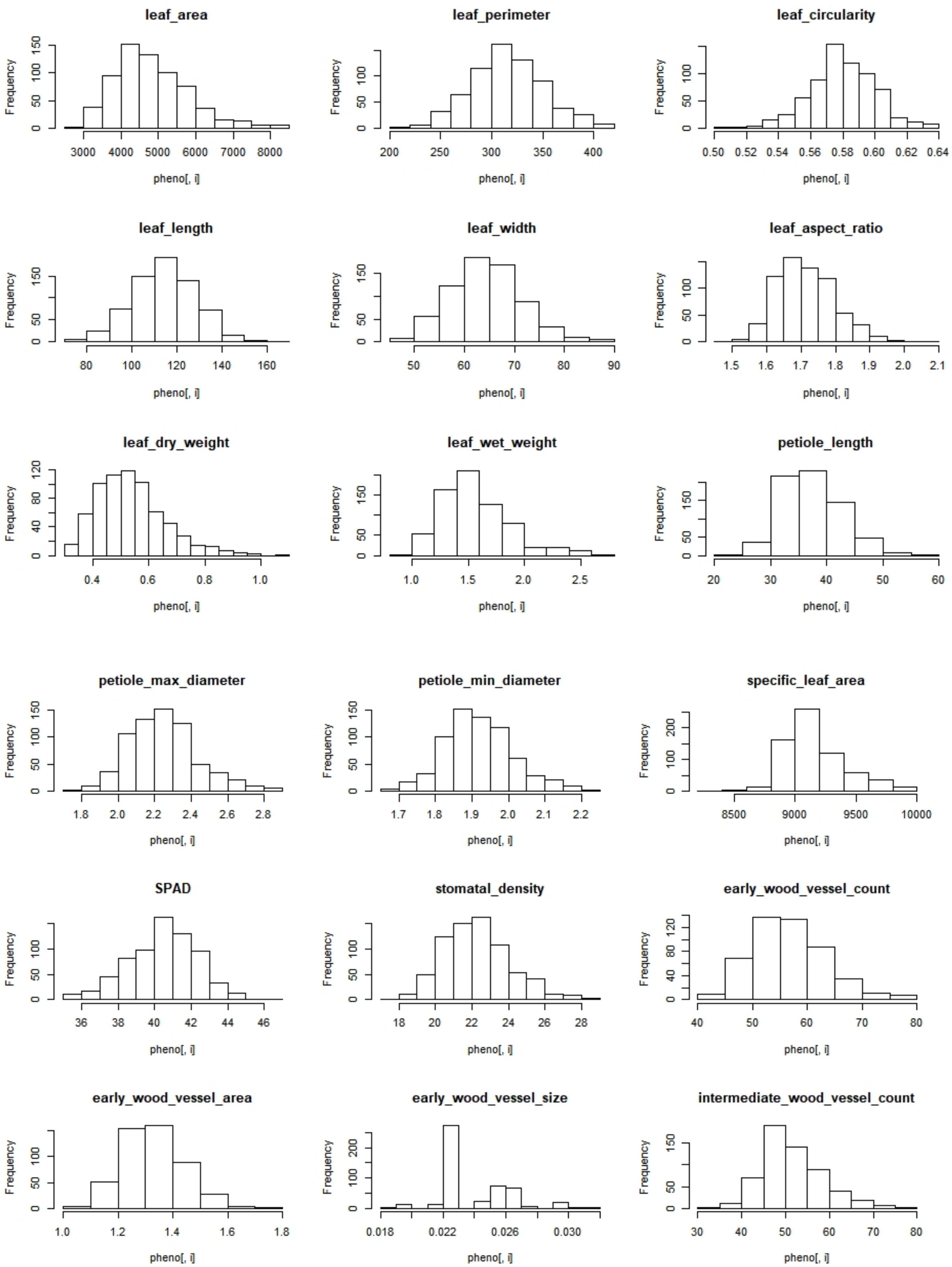
leaf_area
leaf_perimeter
leaf_circularity
leaf_length
leaf_width
leaf_aspect_ratio
leaf_dry_weight
leaf_wet_weight
petiole_length
petiole_max_diameter
petiole_min_diameter
specific_leaf_area
SPAD
stomatal_density
early_wood_vessel_count
early_wood_vessel_area
early_wood_vessel_size
intermediate_wood_vessel_count
Frequency
pheno[, i]

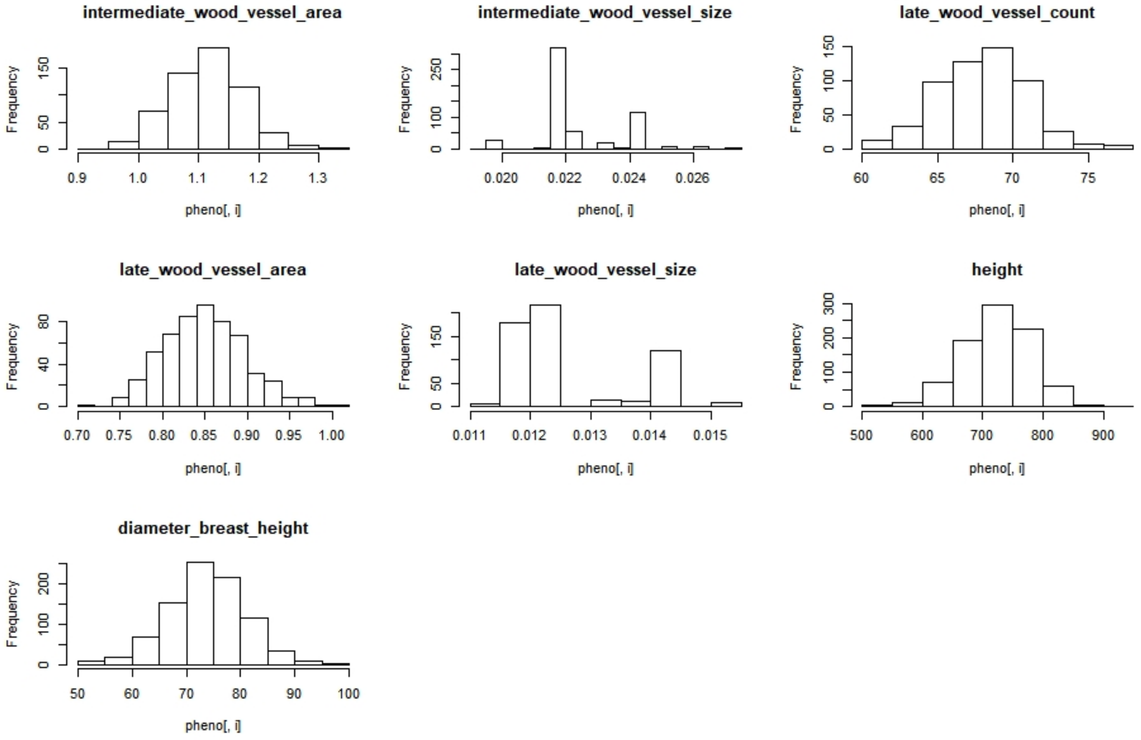

Figure S1. Histograms showing distribution of BLUP adjusted phenotypic values (BLUPs added to the mean phenotypic value) for each of the 25 morphological and anatomical traits used in GWAS study.

Trait																											
Diameter (breast height)	Diameter (breast height)																										
Height	0.816	Height																									
Leaf area	0.028	-0.039	Leaf area																								
Leaf perimeter	0.042	-0.012	0.952	Leaf perimeter																							
Leaf circularity	-0.046	-0.072	0.064	-0.192	Leaf circularity																						
Leaf length	0.031	-0.016	0.898	0.958	-0.243	Leaf length			MORPHOLOGY																		
Leaf width	0.004	-0.057	0.958	0.903	0.179	0.804	Leaf width																				
Leaf aspect ratio	0.060	0.078	-0.244	-0.084	-0.654	0.117	-0.460	Leaf aspect ratio																			
Leaf dry weight	0.006	-0.057	0.894	0.880	-0.039	0.868	0.840	-0.131	Leaf dry weight																		
Leaf wet weight	0.051	-0.022	0.934	0.898	0.014	0.848	0.877	-0.187	0.931	Leaf wet weight																	
Letiole length	-0.064	-0.081	0.601	0.620	-0.054	0.631	0.619	-0.144	0.707	0.566	Letiole length																
Petiole diameter (max)	0.029	-0.047	0.819	0.793	0.029	0.753	0.782	-0.181	0.837	0.880	0.551	Petiole diameter (max)															
Petiole diameter (min)	0.034	-0.032	0.760	0.735	0.030	0.677	0.726	-0.182	0.725	0.828	0.386	0.875	Petiole diameter (min)														
Specific leaf area	0.060	0.027	-0.007	-0.099	0.304	-0.199	0.021	-0.275	-0.322	-0.104	-0.377	-0.117	-0.039	Specific leaf area													
SPAD	0.124	0.105	0.241	0.307	-0.214	0.375	0.215	0.179	0.398	0.279	0.455	0.253	0.148	-0.499	SPAD												
Stomatal density	-0.112	-0.063	-0.198	-0.174	-0.043	-0.157	-0.175	0.028	-0.187	-0.234	-0.058	-0.220	-0.224	-0.045	-0.047	Stomatal density											
Early wood vessel count	-0.065	-0.003	-0.065	-0.058	-0.002	-0.046	-0.060	0.058	-0.081	-0.058	-0.067	-0.062	-0.047	-0.004	-0.057	0.052	0.078	0.037	Early wood vessel count								
Early wood vessel area	0.243	0.266	0.047	0.047	0.030	0.037	0.059	-0.027	0.015	0.041	0.054	0.039	0.013	0.030	0.028	-0.030	-0.020	-0.096	0.557	Early wood vessel area				WOOD ANATOMY			
Early wood vessel size	0.296	0.246	0.059	0.054	0.035	0.018	0.075	-0.113	0.026	0.026	0.066	0.037	0.003	0.102	0.026	-0.049	-0.080	-0.105	-0.432	0.345	Early wood vessel size						
Intermediate wood vessel count	-0.305	-0.217	-0.028	-0.025	-0.015	-0.007	-0.032	0.049	-0.014	-0.011	-0.057	-0.017	-0.010	-0.089	-0.142	0.049	0.140	0.056	0.449	0.023	-0.366	Intermediate wood vessel count					
Intermediate wood vessel area	-0.052	0.018	0.020	0.036	-0.054	0.042	0.022	0.030	0.020	0.040	0.010	0.031	0.006	-0.057	-0.067	-0.013	0.107	0.029	0.268	0.343	0.043	0.610	Intermediate wood vessel area				
Intermediate wood vessel size	0.301	0.231	0.051	0.070	-0.070	0.068	0.039	0.033	0.036	0.062	0.064	0.053	0.021	0.075	0.109	-0.077	-0.106	-0.073	-0.189	0.249	0.408	-0.496	0.175	Intermediate wood vessel size			
Late wood vessel count	-0.231	-0.188	-0.013	-0.017	0.011	-0.013	-0.017	0.017	-0.016	-0.009	-0.019	0.022	0.019	-0.010	-0.148	0.060	0.157	0.072	0.349	0.117	-0.217	0.586	0.399	-0.296	Late wood vessel count		
Late wood vessel area	-0.038	0.045	-0.077	-0.067	-0.020	-0.072	-0.083	0.043	-0.090	-0.038	-0.118	-0.036	0.001	0.047	-0.142	-0.002	0.167	0.025	0.274	0.250	-0.034	0.403	0.531	0.035	0.633	Late wood vessel area	
Late wood vessel size	0.167	0.191	-0.062	-0.056	-0.025	-0.057	-0.077	0.037	-0.073	-0.040	-0.105	-0.071	-0.051	0.064	-0.025	-0.068	-0.010	-0.006	-0.052	0.073	0.145	-0.176	0.016	0.265	-0.426	0.193	Late wood vessel size

Figure S2. Pairwise correlation (r) of *P. trichocarpa* wood anatomical and morphological traits in Clatskanie, Oregon. Shaded values greater than 0.15 or less than -0.15 are significant based on the Bonferroni correction criteria at 5% significance level.

	CORVALLIS																	
	Traits	Water potential	Stomatal density	SPAD	Petiole length	Petiole diameter (max)	Specific leaf area	Leaf area	Leaf perimeter	Leaf length	Leaf width	Leaf feret	Leaf aspect ratio	Leaf dry weight	Carbon isotope	Bud flush	Bud set	Height
CLATSKANIE	Diameter (breast height)	0.09	-0.09	-0.07	0.21	0.17	0.15	0.26	0.28	0.26	0.24	0.25	-0.09	0.18	-0.01	0.23	-0.42	0.38
	Height	0.13	-0.06	-0.05	0.23	0.15	0.14	0.25	0.25	0.22	0.26	0.22	-0.15	0.17	0.00	0.25	-0.45	0.44
	Leaf area	-0.01	-0.12	0.04	0.05	0.09	-0.17	0.10	0.08	0.10	0.07	0.09	0.02	0.14	0.03	-0.15	0.26	-0.15
	Leaf perimeter	-0.01	-0.12	0.07	0.08	0.11	-0.18	0.12	0.11	0.14	0.08	0.13	0.05	0.16	0.05	-0.12	0.26	-0.13
	Leaf circularity	0.01	0.04	-0.06	-0.10	-0.08	0.04	-0.05	-0.09	-0.10	-0.02	-0.10	-0.05	-0.05	-0.04	-0.13	0.03	-0.09
	Leaf length	-0.03	-0.12	0.07	0.07	0.11	-0.18	0.11	0.11	0.15	0.06	0.14	0.07	0.15	0.05	-0.12	0.26	-0.12
	Leaf width	0.00	-0.08	0.04	0.01	0.06	-0.15	0.06	0.04	0.05	0.05	0.04	0.00	0.11	0.02	-0.18	0.29	-0.19
	Leaf aspect ratio	-0.02	-0.07	0.06	0.08	0.09	-0.01	0.09	0.13	0.17	0.03	0.17	0.11	0.06	0.05	0.15	-0.12	0.16
	Leaf dry weight	-0.05	-0.10	0.05	0.02	0.10	-0.23	0.10	0.08	0.10	0.07	0.08	0.01	0.15	0.03	-0.14	0.30	-0.15
	Leaf wet weight	-0.02	-0.14	0.05	0.07	0.15	-0.20	0.17	0.15	0.16	0.13	0.15	-0.02	0.20	0.04	-0.10	0.23	-0.12
	Letiole length	-0.03	-0.02	0.10	0.02	0.01	-0.20	-0.05	-0.05	-0.03	-0.06	-0.04	0.07	0.01	0.01	-0.19	0.40	-0.26
	Petiole diameter (max)	-0.02	-0.12	0.03	0.02	0.13	-0.19	0.12	0.10	0.11	0.11	0.10	-0.03	0.15	0.05	-0.08	0.26	-0.12
	Petiole diameter (min)	-0.01	-0.13	0.04	0.03	0.15	-0.17	0.14	0.13	0.13	0.12	0.12	-0.02	0.16	0.05	-0.05	0.20	-0.10
	Specific leaf area	0.00	-0.03	-0.09	0.02	-0.04	0.09	0.01	0.01	0.02	-0.01	0.02	0.03	-0.02	0.02	-0.02	-0.10	-0.01
	SPAD	-0.05	-0.08	0.20	-0.03	0.01	-0.15	-0.05	-0.05	-0.04	-0.04	-0.04	0.02	-0.01	0.08	0.00	0.20	-0.05
	Stomatal density	-0.02	0.35	-0.09	-0.09	-0.10	0.17	-0.16	-0.16	-0.18	-0.11	-0.18	-0.03	-0.18	-0.09	0.06	-0.01	-0.04
	Early wood vessel count	0.07	-0.03	-0.03	0.03	0.04	0.06	0.04	0.03	0.01	0.05	0.02	-0.03	0.05	-0.01	0.09	-0.13	0.12
	Early wood vessel area	0.08	-0.09	-0.08	0.09	0.09	0.09	0.13	0.13	0.11	0.13	0.11	-0.06	0.13	-0.09	0.07	-0.16	0.15
	Early wood vessel size	-0.02	-0.06	-0.06	0.05	0.04	0.04	0.05	0.07	0.07	0.05	0.06	-0.01	0.05	-0.01	-0.04	-0.03	0.03
	Intermediate wood vessel count	0.06	0.07	0.02	-0.01	-0.01	0.00	0.02	0.00	-0.02	0.01	-0.01	-0.03	0.03	0.01	0.07	-0.05	0.06
	Intermediate wood vessel area	0.06	-0.03	0.01	0.02	0.03	-0.02	0.09	0.09	0.06	0.09	0.07	-0.05	0.09	-0.02	0.10	-0.10	0.12
	Intermediate wood vessel size	-0.06	-0.10	-0.02	0.03	0.06	-0.01	0.09	0.10	0.10	0.09	0.10	-0.01	0.05	-0.02	0.03	-0.11	0.05
	Late wood vessel count	0.05	-0.03	-0.07	0.03	-0.01	0.04	0.02	0.01	0.00	0.02	0.01	-0.01	0.04	0.01	0.06	0.01	0.05
	Late wood vessel area	0.05	-0.05	0.00	0.14	0.10	-0.01	0.13	0.13	0.11	0.13	0.11	-0.06	0.14	-0.05	0.15	-0.16	0.19
	Late wood vessel size	-0.05	-0.02	0.06	0.06	0.04	-0.03	0.06	0.09	0.07	0.06	0.06	-0.04	0.04	-0.02	0.09	-0.16	0.11

Figure S3. Pearson correlation (r) of *P. trichocarpa* wood anatomical and morphological traits in Clatskanie, Oregon with morphological and physiological traits in Corvallis, OR. Shaded values greater than 0.15 or less than -0.15 are significant based on the Bonferroni correction criteria at 5% significance level.

	Trait/Geoclimate	Longitude	Latitude	Elevation	MAT	MWMT	MCMT	TD	MAP	MSP	AHM	SHM	DD_0	DD5	DD_18
MORPHOLOGY	Height	0.346	-0.342	-0.161	0.325	0.119	0.372	-0.386	-0.157	-0.113	0.200	0.057	-0.363	0.266	-0.333
	Diameter (breast height)	0.288	-0.288	-0.128	0.271	0.110	0.316	-0.325	-0.096	-0.073	0.145	0.029	-0.310	0.220	-0.279
	Leaf area	-0.049	0.280	0.148	-0.210	-0.126	-0.198	0.176	0.277	0.345	-0.301	-0.307	0.162	-0.212	0.208
	Leaf perimeter	-0.012	0.268	0.119	-0.184	-0.107	-0.180	0.164	0.225	0.306	-0.258	-0.283	0.148	-0.183	0.182
	Leaf circularity	-0.123	0.081	0.120	-0.127	-0.082	-0.118	0.104	0.120	0.081	-0.121	-0.058	0.102	-0.129	0.129
	Leaf length	-0.004	0.268	0.110	-0.176	-0.114	-0.167	0.145	0.222	0.313	-0.258	-0.294	0.133	-0.179	0.173
	Leaf width	-0.099	0.297	0.166	-0.237	-0.128	-0.232	0.217	0.281	0.331	-0.307	-0.287	0.197	-0.232	0.237
	Leaf aspect ratio	0.158	-0.107	-0.114	0.142	0.037	0.154	-0.168	-0.130	-0.082	0.129	0.037	-0.144	0.124	-0.146
	Leaf dry weight	-0.024	0.302	0.178	-0.230	-0.130	-0.219	0.200	0.310	0.392	-0.329	-0.347	0.181	-0.228	0.228
	Leaf wet weight	0.033	0.233	0.130	-0.161	-0.086	-0.149	0.138	0.290	0.371	-0.297	-0.321	0.115	-0.164	0.159
	Letiole length	-0.200	0.395	0.163	-0.305	-0.186	-0.312	0.286	0.239	0.326	-0.288	-0.295	0.278	-0.288	0.305
	Petiole diameter (max)	0.032	0.240	0.152	-0.178	-0.076	-0.175	0.174	0.302	0.370	-0.306	-0.312	0.140	-0.172	0.178
	Petiole diameter (min)	0.045	0.182	0.108	-0.123	-0.054	-0.118	0.115	0.241	0.305	-0.246	-0.257	0.095	-0.120	0.123
	Specific leaf area	-0.029	-0.057	-0.015	0.032	0.009	0.037	-0.040	0.011	-0.035	0.017	0.054	-0.038	0.021	-0.032
	SPAD	-0.060	0.152	-0.004	-0.084	-0.035	-0.100	0.101	0.016	0.107	-0.052	-0.106	0.082	-0.072	0.083
	Stomatal density	-0.107	-0.157	-0.016	0.048	0.041	0.035	-0.024	-0.107	-0.182	0.138	0.182	0.000	0.069	-0.044
WOOD ANATOMY	Early wood vessel count	0.020	-0.131	-0.042	0.103	0.059	0.103	-0.100	0.025	-0.011	0.012	0.011	-0.099	0.098	-0.104
	Early wood vessel area	0.148	-0.102	-0.015	0.103	0.052	0.115	-0.120	0.024	0.046	0.011	-0.068	-0.132	0.079	-0.106
	Early wood vessel size	0.059	0.031	0.017	-0.007	-0.002	0.002	-0.005	-0.008	0.042	0.006	-0.055	-0.027	-0.025	0.004
	Intermediate wood vessel count	0.043	-0.116	-0.050	0.102	0.109	0.079	-0.045	-0.019	-0.054	0.040	0.079	-0.072	0.114	-0.098
	Intermediate wood vessel area	0.111	-0.122	0.010	0.081	0.064	0.077	-0.065	-0.002	-0.016	0.017	0.009	-0.072	0.078	-0.080
	Intermediate wood vessel size	0.033	-0.012	0.032	-0.001	-0.032	0.021	-0.038	0.002	0.012	0.004	-0.042	-0.011	-0.010	-0.003
	Late wood vessel count	0.067	-0.061	-0.057	0.080	0.042	0.072	-0.066	0.007	0.000	-0.021	-0.031	-0.071	0.075	-0.077
	Late wood vessel area	0.201	-0.219	-0.058	0.183	0.098	0.191	-0.185	-0.050	-0.081	0.088	0.055	-0.185	0.161	-0.184
	Late wood vessel size	0.108	-0.124	-0.029	0.104	0.059	0.119	-0.117	-0.052	-0.082	0.109	0.088	-0.123	0.086	-0.108

	Trait/Geoclimate	DD18	NFFD	bFFP	eFFP	FFP	PAS	EMT	EXT	Eref	CMD	MAR	RH	PC1	PC2	PC3	PC4
MORPHOLOGY	Height	0.135	0.340	-0.261	0.323	0.296	-0.346	0.349	0.153	0.282	0.100	0.116	-0.057	0.320	-0.105	-0.208	-0.153
	Diameter (breast height)	0.107	0.287	-0.224	0.272	0.250	-0.294	0.302	0.128	0.233	0.071	0.103	-0.029	0.265	-0.111	-0.161	-0.143
	Leaf area	-0.165	-0.175	0.124	-0.151	-0.140	0.201	-0.170	-0.232	-0.284	-0.340	-0.074	0.243	-0.244	-0.220	0.160	0.002
	Leaf perimeter	-0.145	-0.154	0.105	-0.133	-0.121	0.164	-0.157	-0.207	-0.255	-0.308	-0.072	0.211	-0.214	-0.195	0.152	0.011
	Leaf circularity	-0.089	-0.127	0.117	-0.119	-0.121	0.138	-0.113	-0.086	-0.113	-0.079	-0.013	0.061	-0.133	-0.011	0.027	-0.012
	Leaf length	-0.145	-0.141	0.095	-0.121	-0.110	0.152	-0.143	-0.216	-0.256	-0.319	-0.065	0.222	-0.208	-0.213	0.139	0.016
	Leaf width	-0.170	-0.210	0.156	-0.185	-0.173	0.233	-0.206	-0.228	-0.296	-0.324	-0.083	0.229	-0.269	-0.184	0.176	0.014
	Leaf aspect ratio	0.060	0.150	-0.124	0.140	0.134	-0.167	0.144	0.054	0.117	0.062	0.049	-0.046	0.145	-0.022	-0.106	-0.005
	Leaf dry weight	-0.168	-0.196	0.137	-0.170	-0.155	0.224	-0.188	-0.243	-0.305	-0.381	-0.051	0.259	-0.268	-0.245	0.199	-0.030
	Leaf wet weight	-0.121	-0.128	0.085	-0.102	-0.095	0.164	-0.122	-0.191	-0.236	-0.347	-0.051	0.235	-0.200	-0.256	0.190	-0.062
	Letiole length	-0.219	-0.280	0.206	-0.267	-0.240	0.278	-0.288	-0.272	-0.354	-0.330	-0.108	0.205	-0.331	-0.138	0.170	0.104
	Petiole diameter (max)	-0.108	-0.155	0.099	-0.129	-0.115	0.191	-0.150	-0.178	-0.240	-0.340	-0.033	0.215	-0.215	-0.231	0.225	-0.081
	Petiole diameter (min)	-0.070	-0.101	0.055	-0.077	-0.067	0.136	-0.098	-0.141	-0.184	-0.280	-0.048	0.178	-0.155	-0.205	0.178	-0.067
	Specific leaf area	-0.004	0.025	0.006	0.023	0.007	-0.018	0.028	0.034	0.051	0.055	-0.009	-0.039	0.029	0.022	-0.040	-0.029
	SPAD	-0.053	-0.073	0.020	-0.077	-0.048	0.041	-0.090	-0.088	-0.117	-0.112	-0.077	0.073	-0.088	-0.047	0.072	0.105
	Stomatal density	0.079	0.011	0.024	0.002	-0.011	-0.016	0.020	0.139	0.154	0.216	0.030	-0.223	0.073	0.201	-0.072	-0.043
WOOD ANATOMY	Early wood vessel count	0.080	0.090	-0.056	0.103	0.080	-0.057	0.093	0.089	0.121	0.045	0.015	-0.059	0.091	-0.025	-0.005	-0.090
	Early wood vessel area	0.044	0.111	-0.087	0.121	0.104	-0.093	0.114	0.035	0.073	-0.040	0.086	0.041	0.087	-0.110	-0.014	-0.090
	Early wood vessel size	-0.043	0.010	-0.021	0.002	0.013	-0.031	0.011	-0.048	-0.045	-0.061	0.039	0.092	-0.009	-0.061	-0.012	0.030
	Intermediate wood vessel count	0.129	0.078	-0.048	0.084	0.069	-0.057	0.078	0.136	0.133	0.091	0.026	-0.090	0.099	0.039	0.053	-0.080
	Intermediate wood vessel area	0.072	0.067	-0.029	0.076	0.053	-0.053	0.073	0.082	0.107	0.038	0.044	-0.067	0.075	-0.002	0.004	-0.136
	Intermediate wood vessel size	-0.031	0.013	-0.017	0.019	0.018	-0.004	0.018	-0.047	-0.013	-0.026	0.011	0.027	-0.001	-0.036	-0.055	-0.012
	Late wood vessel count	0.057	0.076	-0.063	0.084	0.074	-0.056	0.071	0.050	0.065	-0.003	-0.031	-0.020	0.066	-0.047	0.016	-0.053
	Late wood vessel area	0.106	0.171	-0.120	0.170	0.146	-0.159	0.175	0.137	0.193	0.094	0.049	-0.096	0.177	-0.014	-0.067	-0.158
	Late wood vessel size	0.048	0.104	-0.075	0.096	0.086	-0.098	0.111	0.069	0.102	0.086	0.073	-0.025	0.110	0.013	-0.073	-0.046

Figure S4. Pearson correlation (r) of *P. trichocarpa* wood anatomical and morphological traits in Clatskanie, Oregon with geo-climate variables of the source location of the trees. Shaded values greater than 0.15 or less than -0.15 are significant based on the Bonferroni correction criteria at 5% significance level.

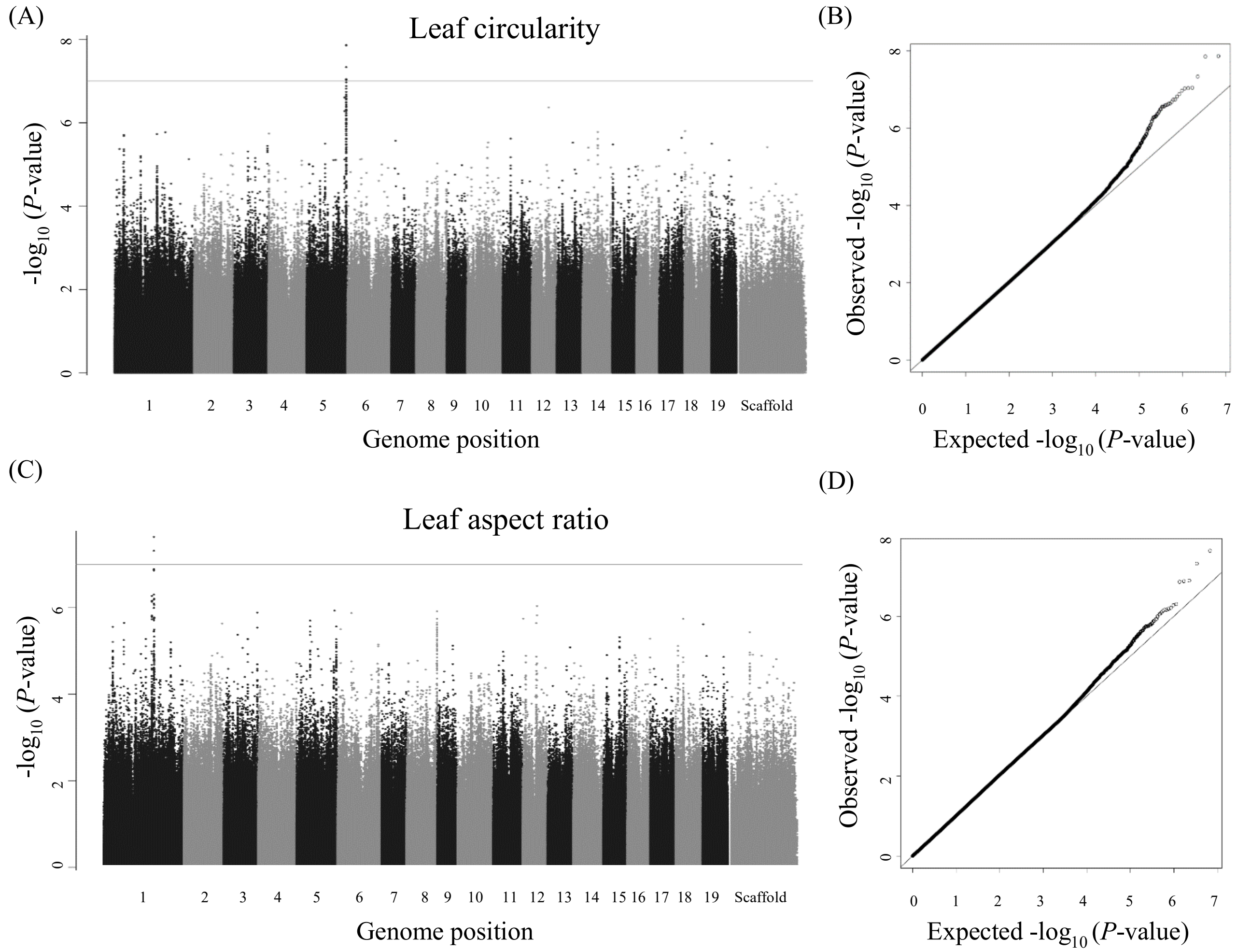

Figure S5. Single trait GWAS – Manhattan (left) and QQ plots (right). Numbers 1 to 19 represent chromosomes; scaffolds are the reads that did not align to any of the 19 chromosomes. (A) and (B) Leaf circularity; (C) and (D) Leaf aspect ratio. SNPs above gray line have $p<1\times10^{-7}$, which is roughly equivalent to FDR≤0.1 and SNPs above black line (the top line) have $p<7.417\times10^{-9}$ (the Bonferroni correction threshold), which is roughly equivalent to FDR≤0.05 in this study.

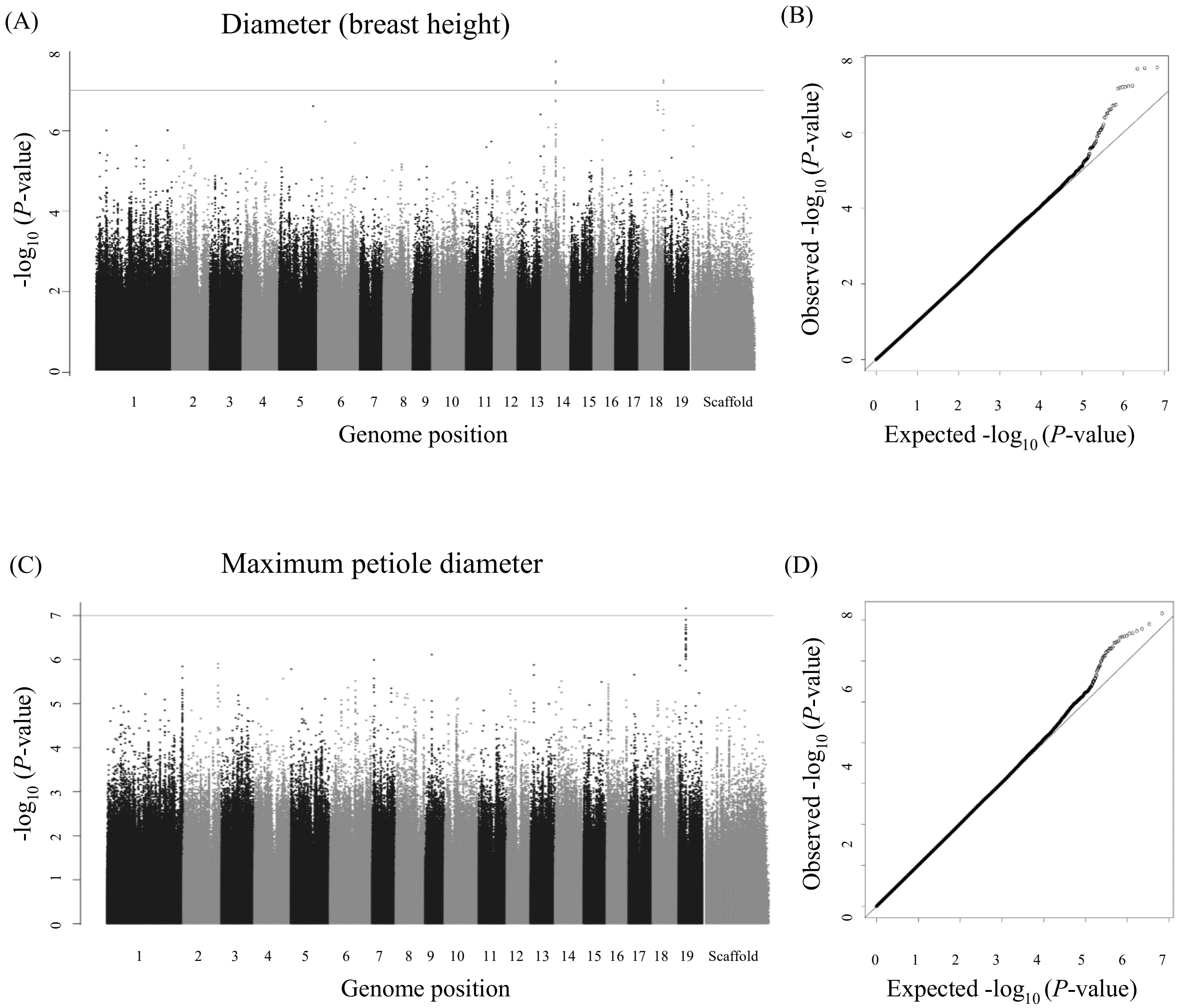

Figure S6. Single trait GWAS – Manhattan (left) and QQ plots (right). Numbers 1 to 19 represent chromosomes; scaffolds are the reads that did not align to any of the 19 chromosomes. (A) and (B) Diameter (breast height); (C) and (D) Maximum petiole diameter. SNPs above gray line have $p<1\times10^{-7}$, which is roughly equivalent to FDR≤0.1 and SNPs above black line (the top line) have $p<7.417\times10^{-9}$ (the Bonferroni correction threshold), which is roughly equivalent to FDR≤0.05 in this study.

(A) Vessel area (early wood), leaf area and stomatal density (B)

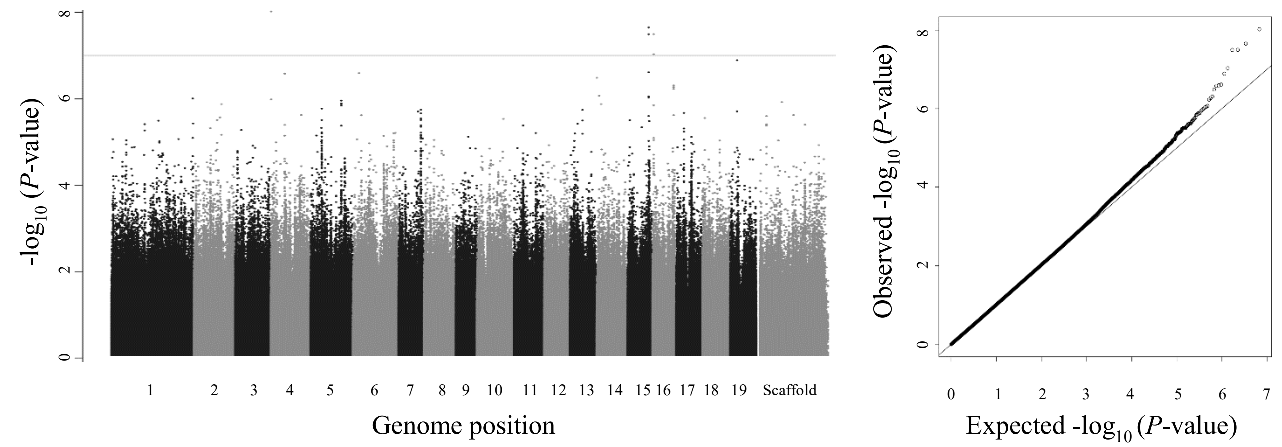

Figure S7. Multitrait GWAS – Manhattan (left) and QQ plots (right). Numbers 1 to 19 represent chromosomes; scaffolds are the reads that did not align to any of the 19 chromosomes. (A) and (B) Vessel area (early wood), leaf area and stomatal density. SNPs above gray line have $p<1\times10^{-7}$, which is roughly equivalent to FDR≤0.1 and SNPs above black line (the top line) have $p<7.417\times10^{-9}$ (the Bonferroni correction threshold), which is roughly equivalent to FDR≤0.05 in this study.

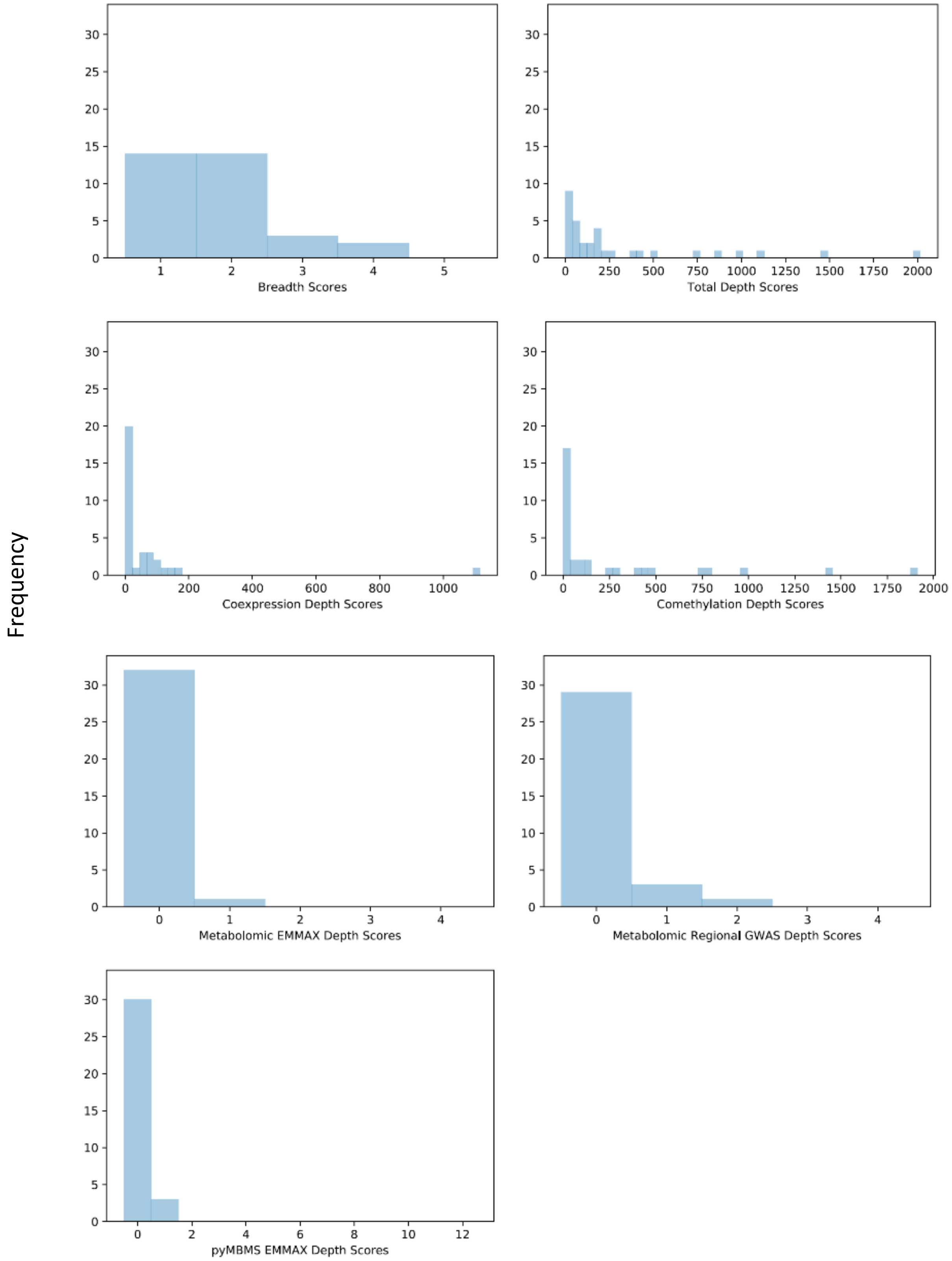

Figure S8. Distributions of LOE scores for all single trait and multitrait anchor (GWAS) genes.

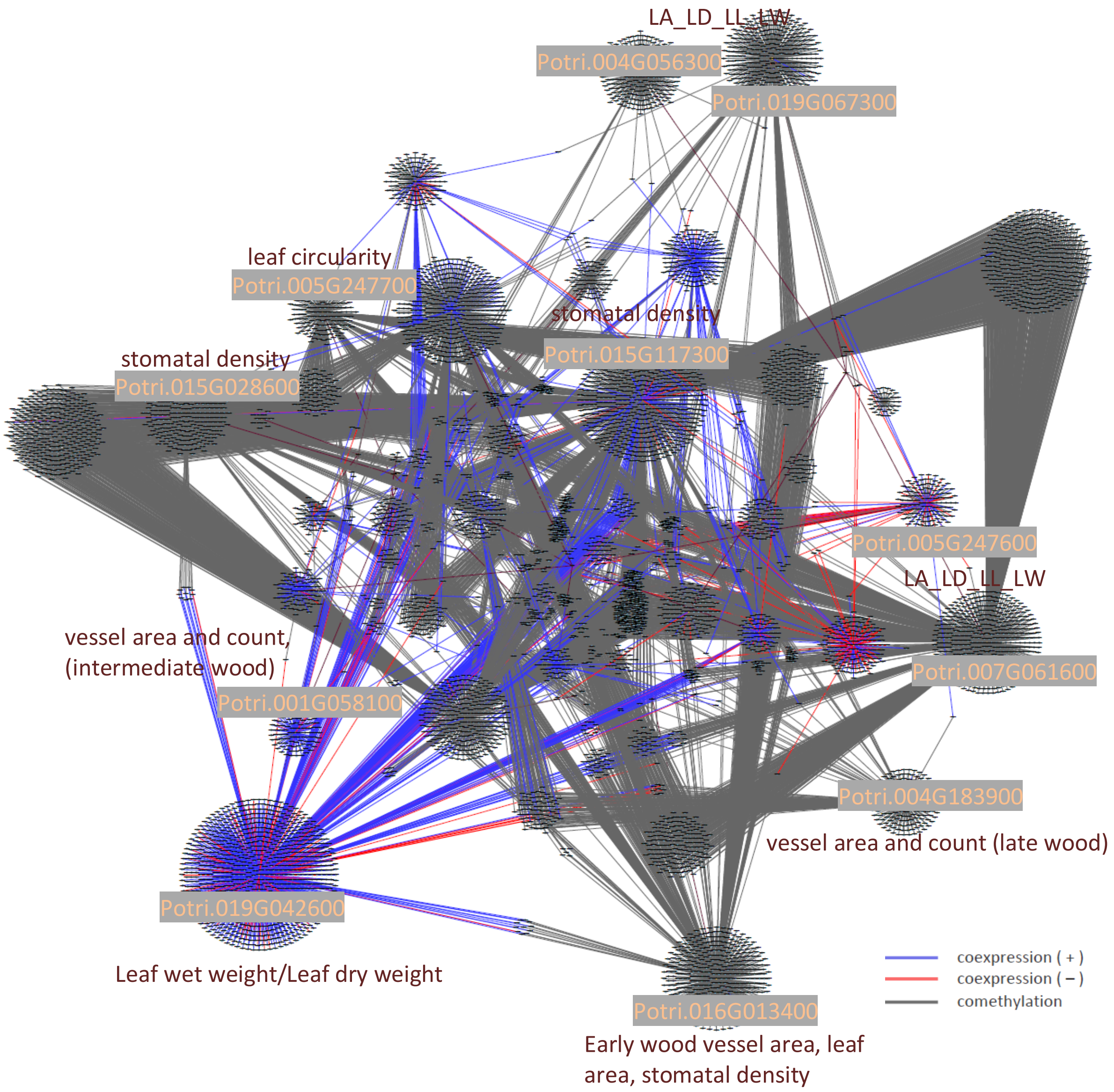

Figure S9. One-hop multi-omic network for all single and multitrati GWAS (anchor) genes with major anchor genes highlighted. LA_LD_LL_LW = leaf area, leaf dry weight, leaf length and leaf width. For GWAS edges, solid maroon indicates "traditional" GWAS and dashed maroon indicates RV metabolite GWAS.

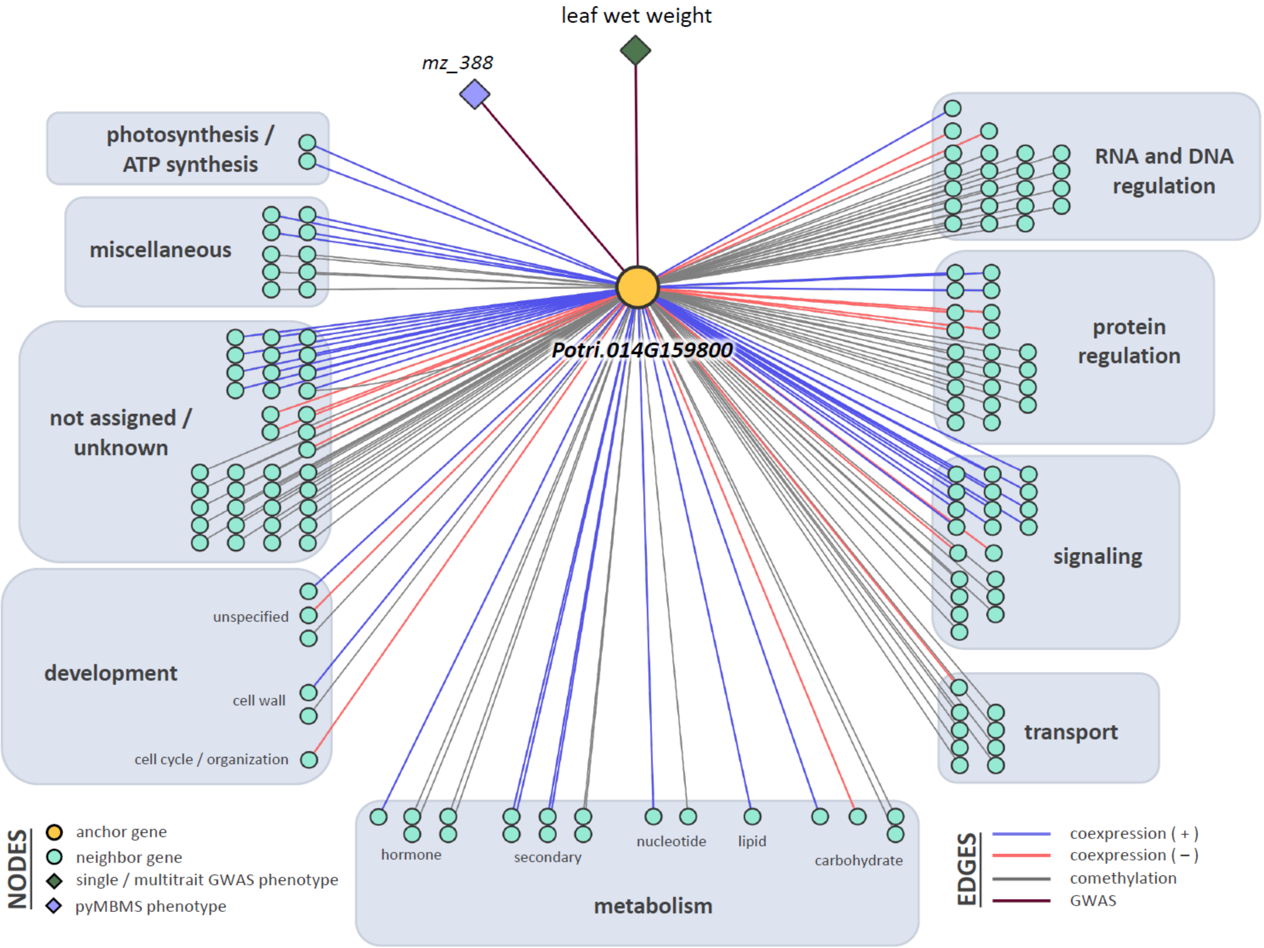

Figure S10. One-hop multi-omic network for the gene Potri.014G159800, which was associated with leaf wet weight.

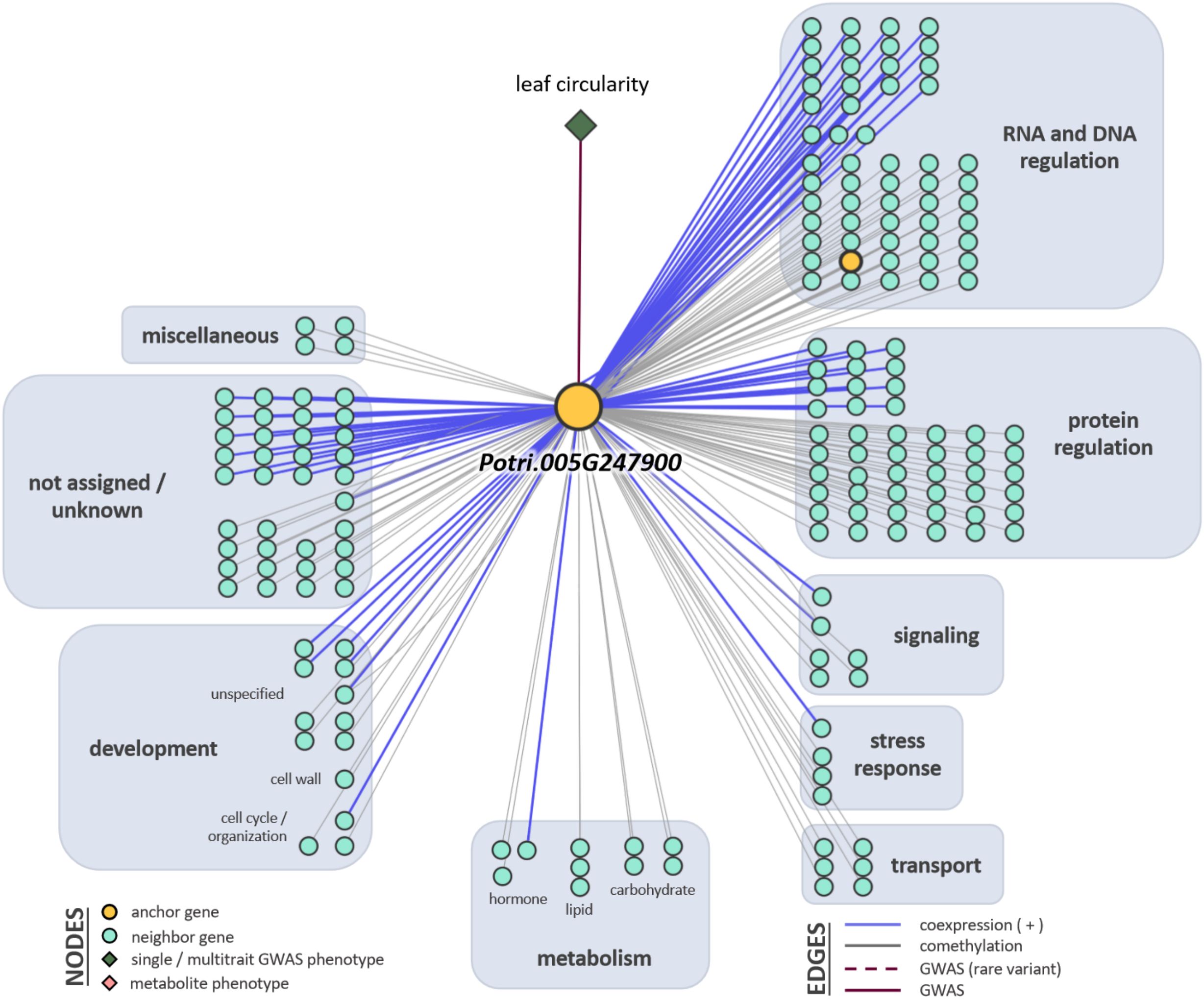

Figure S11. One-hop multi-omic network for the gene Potri.005G247900, which was associated with leaf circularity.

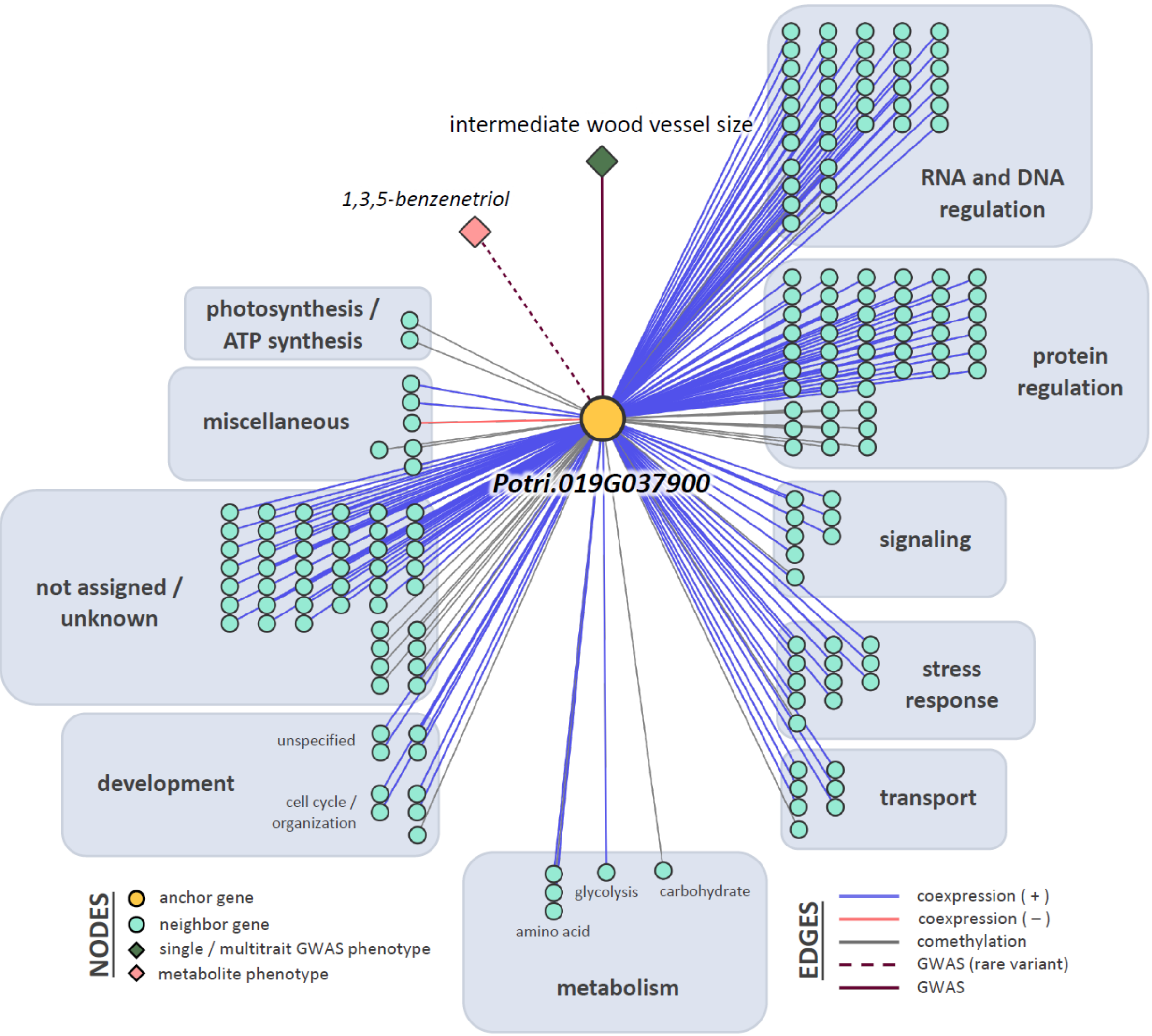

Figure S12. One-hop multi-omic network for the gene Potri.019G037900, which was associated with intermediate wood vessel size.

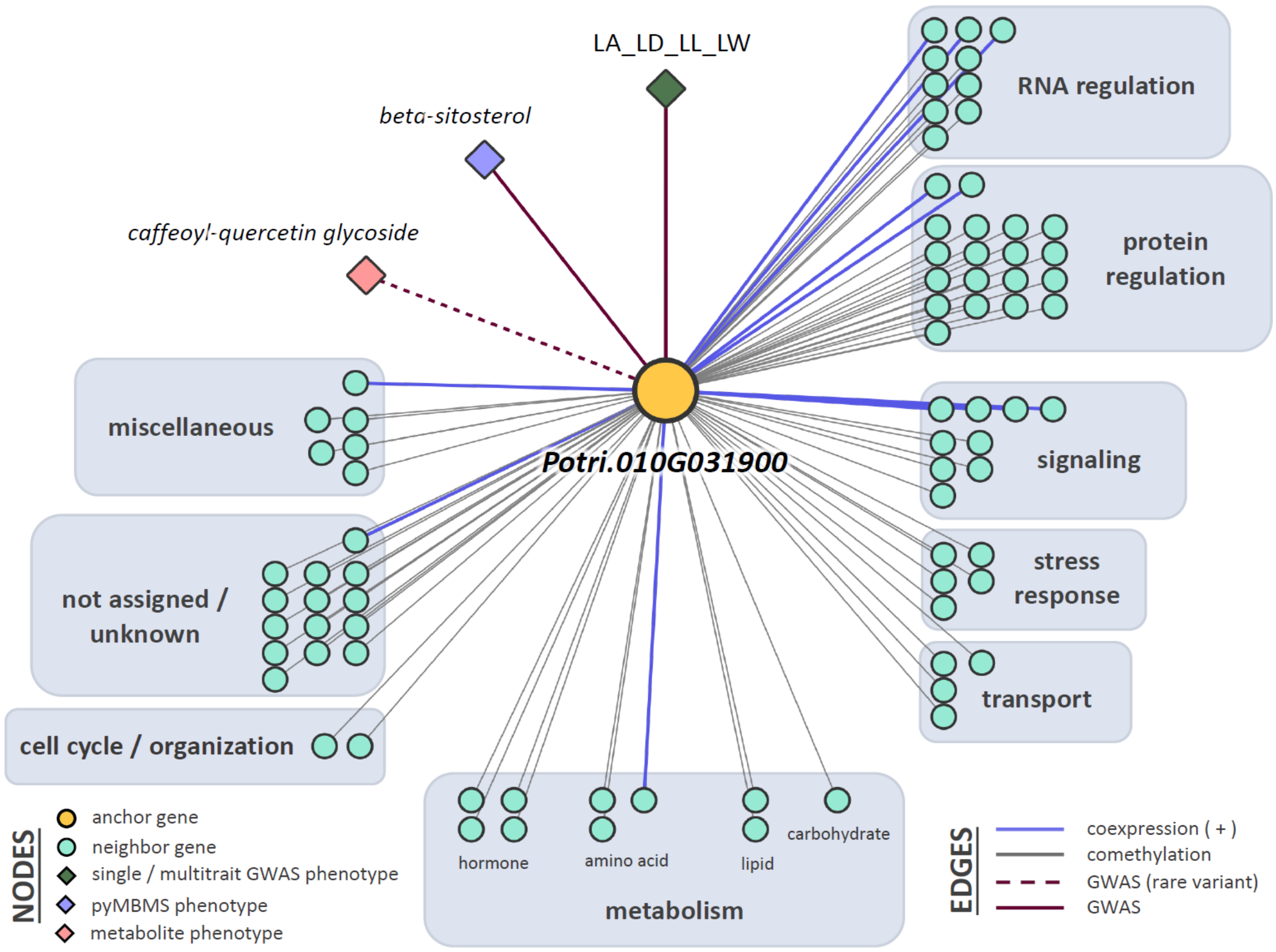

Figure S13. One-hop multi-omic network for the gene Potri.010G031900, which was associated with the multitrait phenotype leaf area, leaf dry weight, leaf length and leaf width.

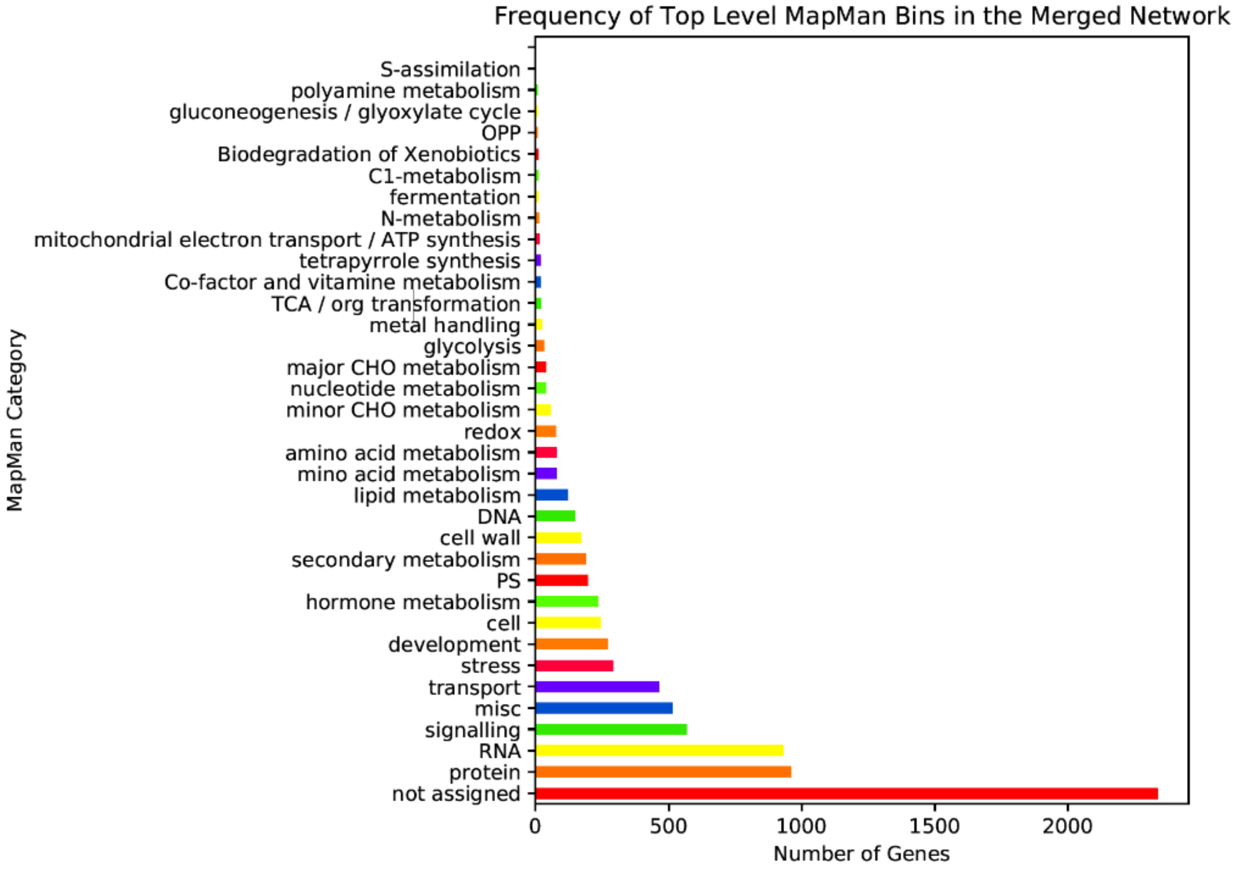

Figure S14. Mapman annotations for all single trait and multitrait GWAS (anchor) genes.

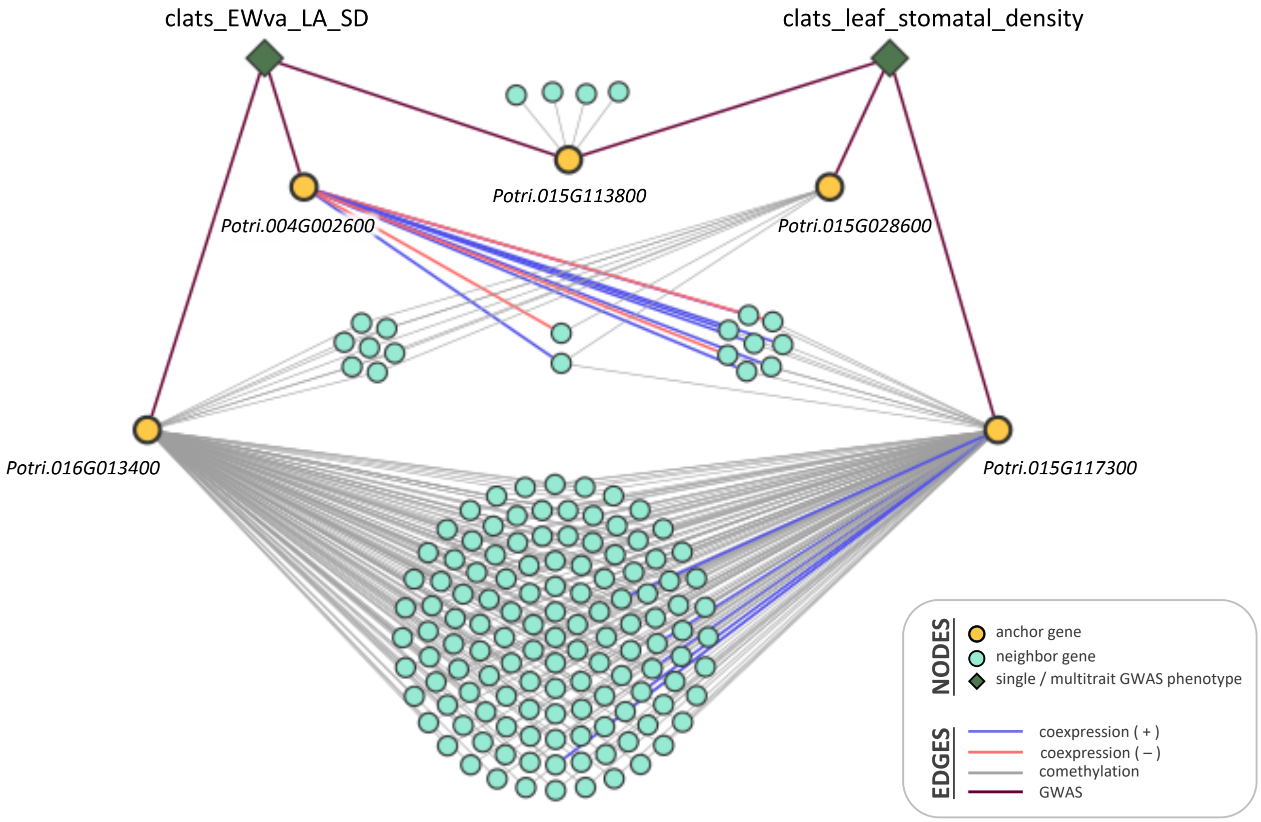

Figure S15. Intersections of subnetworks for single-trait GWAS for Stomatal density and multi-trait GWAS for Early Wood vessel area, Leaf Area, and Stomatal Density.
